# Global invasive alien plant management lists: Assessing current practices and adapting to new demands

**DOI:** 10.1016/j.pld.2024.11.002

**Published:** 2024-11-14

**Authors:** Fei-Fei Li, Qiang Hao, Xia Cui, Ruo-Zhu Lin, Bin-Sheng Luo, Jin-Shuang Ma

**Affiliations:** a Beijing Botanical Garden, Beijing 100093, China; b Key Laboratory of National Forestry and Grassland Administration on Plant Ex situ Conservation, Beijing 100093, China; c Key Laboratory of Forest Protection of the National Forestry and Grassland Administration, Institute of Forest Ecology, Environment and Nature Conservation, Chinese Academy of Forestry, Beijing 100091, China; d Lushan Botanical Garden, Jiangxi Province and Chinese Academy of Sciences, Lushan 332900, China

**Keywords:** Invasive alien plants, Kunming-Montreal global biodiversity framework, Global management strategies, Tiered classification system, Biodiversity conservation

## Abstract

Invasive alien species (IAS) significantly threaten global biodiversity and ecosystem stability. Despite increasing management efforts, a critical knowledge gap existed in understanding commonalities and disparities among national strategies. We analyzed several IAS management lists from 23 countries and the European Union, focusing specifically on vascular plant species within these lists. List composition, characteristics, and associated management measures were analyzed. Key patterns in species prioritization across national lists and intercontinental exchange of invasive alien plants (IAPs) were identified. *Pistia stratiotes*, *Pontederia crassipes*, *Salvinia molesta*, *Cabomba caroliniana*, *Ulex europaeus* were identified as globally recognized threats, being listed by at least 33.3% of analyzed countries and invading five or more continents. Aquatic plants were found to be more frequently included in management lists. A significant directional invasion pattern between the Eastern and Western Hemispheres was identified. Species native to Asia were observed to dominate as significant donors of IAPs across continents. The analysis of list management strategies highlighted substantial gaps in achieving Target 6 of the Kunming-Montreal Global Biodiversity Framework, particularly in species prioritization and inclusion of potential IAPs. In response to these challenges, a tiered classification system for invasive alien species list was proposed, encompassing High-Priority, Watchlist, Potential, and Priority Site categories, which aimed at enhancing management effectiveness by tailoring strategies to different invasion stages and ecological contexts. This study could contribute to understanding global IAPs management strategies and serve as a reference for policymakers and conservation managers to identify priority IAPs and refine management approaches.

## Highlights

• Five globally recognized invasive alien plants (IAPs) identified, listed by ≥33.3 % of analyzed countries.
• Aquatic plants more likely to be included in IAPs management lists.
• Significant directional invasion pattern observed between Eastern and Western Hemispheres.
• Tiered classification system proposed to enhance IAS management effectiveness.

## Graphical abstract

**Figure:**
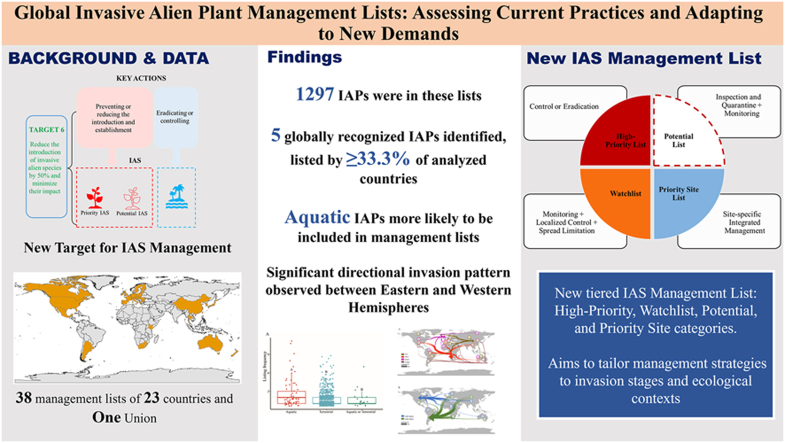

## 1 Introduction

Invasive alien species (IAS) have become one of the greatest threats to global biodiversity and ecosystem stability (Pyšek et al., 2012, 2020). Since Charles Elton first drew attention to biological invasions in his book “The Ecology of Invasions by Animals and Plants” (Elton, 1958), there has been growing evidence that IAS are displacing native species (Winter et al., 2009; Vilà et al., 2011; Pyšek et al., 2012; Cameron et al., 2016), altering community structure, and damaging ecosystem functions worldwide (Gaertner et al., 2014; Capinha et al., 2015; Vilà and Hulme, 2017).

Over the past few decades, efforts have been made globally across various dimensions, such as prevention, risk assessment, and control management of IAS (Bertolino et al., 2020; Booy et al., 2020; Hulme, 2020; Venette et al., 2021). However, the continuing growth of IAS shows that the threat from IAS keeps rising. Expansions in global trade, tourism, agriculture, horticulture, and transportation networks have provided more opportunities for non-native species to be introduced into new ecosystems (Hanspach et al., 2008; Hulme, 2009; Pyšek et al., 2020). The rate of first records of alien species worldwide has grown over the past 200 years, with 37% of all initial recordings occurring most recently between 1970 and 2014 (Seebens et al., 2017).

Comprehensive and up-to-date species lists are crucial for developing effective strategies to address and manage IAS (Pagad et al., 2018; Seebens et al., 2015). Detailed information on the taxonomy, origins, ecological impacts, and current distribution of alien species can assist governments in tracking the influx of non-native species, identifying high-risk introduction pathways, and prioritizing management based on threat levels (Lieurance et al., 2023). Efforts were made to document and assess the status of alien species invasions worldwide. Databases such as the Global Register of Introduced and Invasive Species (GRIIS, https://griis.org/, consulted in September and December 2023) currently provided information on almost 23,700 species introduced and IAS across 196 countries (Pagad et al., 2022). And the CABI Invasive Species Compendium (CABI ISC) provided detailed coverage of 8409 taxa of invasive pests, plants, fungi, and animal diseases (https://www.cabidigitallibrary.org, consulted in March 2024).

The challenges posed by the large number of IAS are management issues, particularly regarding which species should be prioritized for management (McGeoch et al., 2016). While global databases such as GRIIS and CABI ISC provide valuable information on the distribution and impacts of IAS, uniform management approaches are impractical due to limited financial, human resources and varying levels of species-specific harm. On a national and international level, many countries and organizations have taken actions to address the challenges of IAS by developing relevant policies and plans. The Aichi Targets proposed identifying and prioritizing IAS by 2020 (Aichi Target 9) (McGeoch et al., 2016; Booy et al., 2020). To establish an early warning and rapid response system, the European Union (EU) introduced the Invasive Alien Species Regulation (Regulation (EU) 1143/2014) and has been continuously updating its list of IAS (Regulation (EU) 2022/1203). Similarly, the United States National Invasive Species Management Plan identified critical strategies for prevention, monitoring, and control (National Invasive Species Council, 2016).

These prioritizing IAS lists are often influenced by local research, specific national priorities, and regulatory frameworks. Researchers have conducted in-depth discussions on the identification, risk assessment, and prioritization of IAS. For example, Blackburn et al. (2014) proposed a unified classification framework for IAS, providing a theoretical basis for determining management priorities. Kumschick et al. (2012) developed a multi-criteria decision support tool for assessing and prioritizing the management of IAS. Additionally, some studies focused on prioritizing IAS for specific taxonomic groups or regions, such as alien plants (Weber et al., 2009) and marine IAS (Molnar et al., 2008). In 2000, the Invasive Species Specialist Group (ISSG) of the International Union for Conservation of Nature (IUCN) published “100 of the World's Worst Invasive Alien Species”, which included 32 terrestrial plants and 4 aquatic plants (Lowe et al., 2000), and the latest list currently comprises 38 plants (Luque et al., 2014). Though not fully representative of global invasion profiles, this seminal list marked an attempt towards prioritization of widespread invaders for guiding mitigation globally across habitats (Cuthbert et al., 2022).

Developing national-level management lists and corresponding management strategies for IAS became an increasingly common practice. However, several issues still persisted. García-de-Lomas and Vilà (2015) summarized four main points of criticism regarding management lists: (1) unlisted organisms might pose unknown risks (Simberloff, 2006; Fowler et al., 2007; Brasier, 2008); (2) the slow update rate of harmful species lists (Brasier, 2008); (3) the difficulty of national-level lists in addressing the mismatch between species' natural distributions and the political boundaries to which the lists applied (Simberloff, 2006); and (4) legislative differences among neighboring countries (García-de-Lomas and Vilà, 2015). In addition to these issues, the number of listed species varies significantly between countries, and excessive listing can lead to management burdens. There were few international guidelines indicating the proportion of a country's IAS that should be included in a national list or why certain species were prioritized for management among the numerous IAS within a country (García-de-Lomas and Vilà, 2015). The commonalities and disparities among national lists and management strategies may reflect the inherent harmfulness of the IAS themselves, and differences in the identification and management objectives for IAS. However, few studies thoroughly analyzed the similarities and differences among these lists or the comprehensiveness of management strategies. Clarifying these issues is crucial for developing more targeted and consistent management approaches.

COP15 adopted the new Kunming-Montreal Global Biodiversity Framework. A more ambitious and quantified target related to IAS was established. Target 6, which aims to eliminate, minimize, reduce, and or mitigate the impacts of IAS on biodiversity and ecosystem services by 2030, identifies three categories: (1) priority IAS; (2) potential IAS; and (3) IAS in priority sites such as islands. It also emphasizes two key actions: (1) preventing or reducing the introduction and establishment; (2) eradicating or controlling. The differentiated categorization under Target 6 and the gaps constraining its implementation poses new challenges for establishing national and international management strategies and lists.

Considering these challenges, this study focuses on invasive alien plants (IAPs) and aims to provide insights addressing key knowledge barriers, and provide insights for broader IAS management strategies while offering global references to support the achievement of Target 6. IAPs were selected as the focal group due to their predominance among IAS and their status as the most comprehensively studied taxonomic group (Pyšek et al., 2008). Furthermore, the existence of comprehensive IAP lists and management strategies in many countries provides rich data for comparative analysis (Turbelin et al., 2017). Based on IAPs from management lists of multiple countries or alliances worldwide, this research provides insights to answer the following questions: Which categories of IAPs pose widespread threats and are of common concern to multiple countries? Do these national management lists exhibit certain biases? Do the existing management approaches associated with these lists align with the objectives of Target 6?

## 2 Material and methods

### 2.1 Data selection

Only government-issued species lists were considered in this study. IAPs included in this study were vetted across the following aspects: the species list must originate from union or national-level agencies, such as those responsible for the environment, agriculture, forestry, customs, or related domains, and be issued as published records or legal instruments. Documents from sub-national territories within continental landmasses were excluded, given varied invasion pressures across provinces and potentially inconsistent coordination of biodiversity policies countrywide. However, these were included in our analysis for countries with overseas territories or areas due to their unique ecosystems, distinct invasion risks, and integral administrative status within their respective nations. As Tracheophyta (vascular plants) species account for the vast majority of terrestrial and aquatic invasive records impacting ecosystems and human systems, only species under this taxonomic group were selected in this study.

Our primary sources were the 6th National Reports submitted to the Secretariat of the Convention on Biological Diversity (CBD) by member countries worldwide (https://chm.cbd.int/search/reporting-map?filter=nr6, consulted from May to August 2023), the ECOLEX database which provides access to environmental laws and policies including those related to invasive species management (https://www.ecolex.org/, consulted from May to August 2023), and the previous study of García-de-Lomas and Vilà (2015). Utilizing these data sources, we conducted a comprehensive search across 105 countries. Based on the criteria outlined above, we identified a total of 38 species lists that met our selection requirements (Table S1). These lists originate from 23 individual countries and the European Union (EU), spanning six continents. In our analysis, the EU list was treated as a single entity. Additionally, we included separate national lists from individual EU member states if they had established their own additional lists beyond the EU-wide list. These national lists were treated as independent lists belonging to their respective countries. For clarity and consistency throughout this paper, we use the term “countries” to refer collectively to the 23 individual nations and the EU, unless specifically stated otherwise.

### 2.2 Data standardization and information related

To enable accurate categorization and traceability of species policy statuses across countries, scientific names documented in lists were standardized according to the Plants of the World Online database (POWO, https://powo.science.kew.org/, consulted in September and October 2023), which served as the primary standard for ensuring uniformity. This process involved consolidating outdated synonyms, ensuring a unified compilation of global figures on genera and families across sources.

Throughout this study, we used the term “species” broadly to encompass species, subspecies, varieties, and other taxonomic ranks (including “spp.” notations) within vascular plants. When exact taxonomic precision is required, we used the term “taxa”. In some lists, taxonomic groups were represented using the genus name followed by “spp.” to indicate multiple species within that genus. When calculating the number of countries that had listed each species, we employed the following approach. If the total species list did not include any specific species within a genus, we retained the “spp.” entry as a species. Otherwise, if a country's list included a genus name followed by “spp.”, all species within that genus in the total species list were considered included in that country's list, excluding any species native to that country. This approach ensures that the presence of “spp.” in a country's list was interpreted as including all non-native species within that genus, without explicitly listing each species.

Species habitats were categorized into three types: aquatic, aquatic or terrestrial, and terrestrial. Growth forms classifications were based on criteria from Engemann et al. (2016), dividing species into seven categories: epiphyte, herb, liana, parasitic, shrub, tree, and vine. In cases where a species was classified as both a tree and a shrub, we adopted the tree classification. Species data for habitats and growth forms classification were predominantly obtained from the POWO and The World Flora Online (WFO, https://www.worldfloraonline.org/, consulted in September and December 2023). Information on species’ native distribution at the continental level was also extracted from the POWO.

The statistical data on introduced and invasive plants (including only vascular plants) within various countries were sourced from the GRIIS. The species for the EU encompasses the data from all 27 EU member states as recorded in GRIIS, with duplicate species not counted in the final tally.

### 2.3 Species data analysis

Basic statistical analyses were performed using Excel. To investigate potential biases in species selection for national management lists, we employed a Poisson regression model using the “*LME4*” package (Bates et al., 2014) in R (R Core Team, 2022). This model analyzed the relationship between the independent variables (habitats, growth forms, and native range) and the dependent variable (listing frequency of IAPs). Native range was quantified as the number of continents in which a species naturally occurs. Additionally, pie charts illustrating the proportion of each habitat type and growth form in various countries' lists were created using ArcGIS 10.4. Species similarities between all countries were calculated using the “jac_mat” function from the “*vegan*” package (Oksanen et al., 2010) in R project. To assess the directionality of species exchange between continents and between the Eastern and Western hemispheres, we conducted McNemar's chi-squared tests using R project. For this analysis, we defined the Eastern and Western hemispheres based on continental groupings. The Eastern hemisphere is comprised of Asia, Africa, Europe, and Oceania, while the Western hemisphere is composed of North America and South America. For countries with territories spanning multiple continents, we classified overseas territories and dependencies according to their geographic location rather than political affiliation. French Guiana was classified as part of South America, while Saint Martin, Martinique, and Guadeloupe were considered part of North America. French Reunion, French Mayotte, and Spain's Canary Islands were classified as part of Africa. This decision was made to more accurately reflect the ecological and biogeographical context of invasive species spread and management.

### 2.4 Classification and analysis of list management strategies

**Fig. 1:**
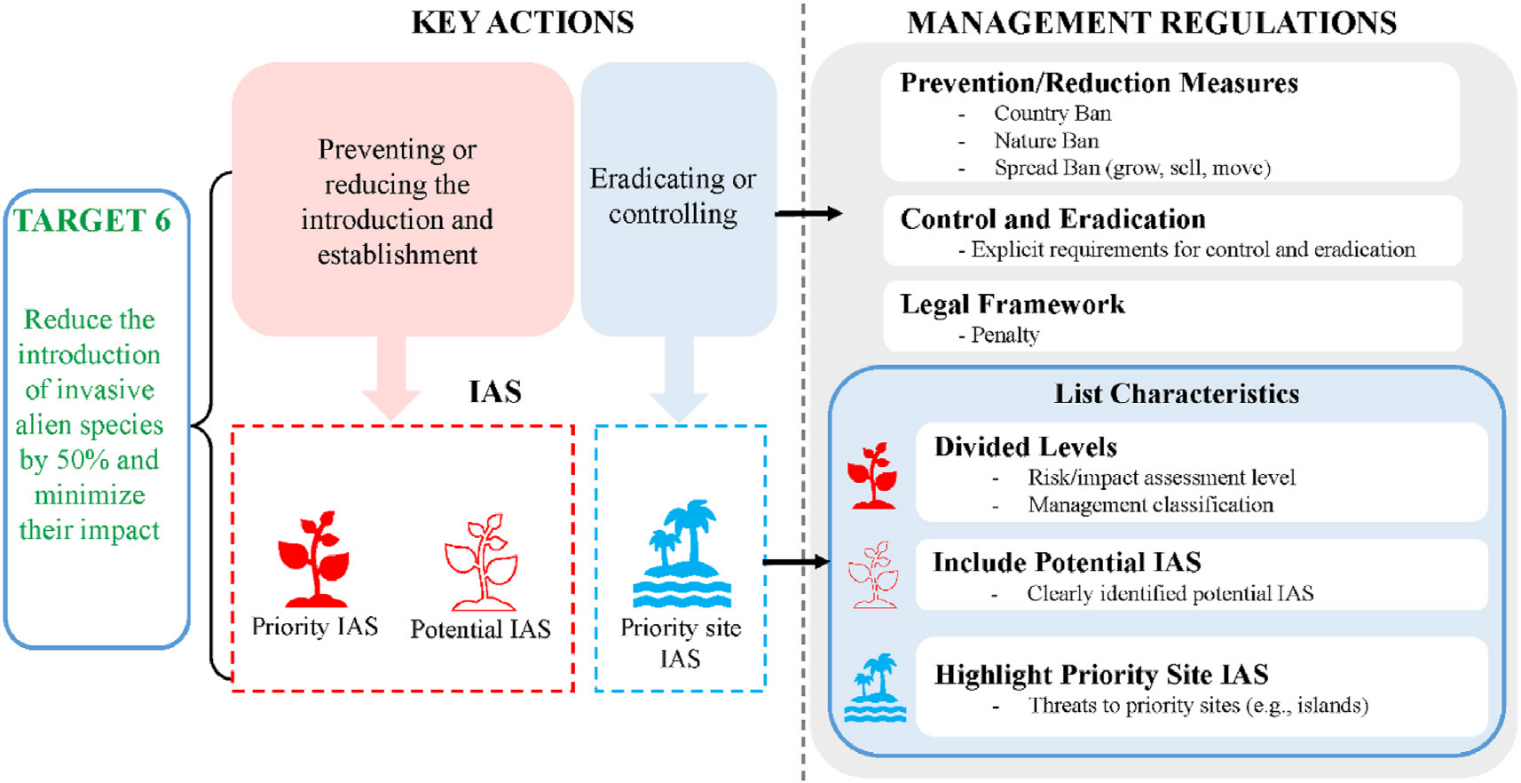
Framework for managing invasive alien species (IAS) under Target 6.

Based on the key actions outlined in Target 6, we focused on three primary characteristics each for lists and management regulations (Fig. 1). For lists, the main characteristics include: (1) whether IAPs in the lists of the country were divided levels (Divided Levels), including risk/impact assessment level and management classification; (2) whether the lists of the country contained potential IAPs clearly; (3) whether the lists of the country highlights IAPs that threaten priority sites, including lists highlights IAPs that threaten priority sites and lists that were developed specifically for priority sites (such as islands). For management regulations associated with these lists, we examined: (1) the inclusion of measures to prevent or reduce IAPs introduction and establishment, specifically analyzing regulations for forbidden introduction into the country (Country Ban), natural ecosystem/environment management (Nature Ban), and prohibited spreading (Spread Ban, including prohibitions on growing, selling, and moving); (2) explicit requirements for control and eradication of IAPs (Control); and (3) the legal framework supporting list management, specifically assessing whether the lists originated from legal documents (encompass various normative documents formulated by legislative or administrative authorities, including laws, regulations, rules, and ordinances) or official lists (informational or strategic documents compiled by government agencies or authorized institutions, which may not have direct regulatory power but serve as official guidance), and whether they incorporated clear legal penalties (Penalty).

To investigate the impact of management strategies on species lists across countries, we conducted a cluster analysis of national management policies and correlation analysis between these policies and the number of IAPs in lists. We utilized finer-grained management strategy indicators, including Country Ban, Nature Ban, Spread Ban, Control, Penalty, and Divided Levels. These approaches were chosen to capture the subtle differences in policy implementation among countries and to provide a more comprehensive understanding of how specific management strategies might influence the size of IAPs lists. Our primary aim was to examine the direct, operational aspects of IAPs management rather than the legislative processes behind them. To this end, we excluded “legal document origin” as a separate indicator to avoid redundancy, given that many of our indicators likely derive from legal documents, and to account for varying legal systems across countries. This approach allowed us to isolate the effects of specific management actions on IAPs lists, focus on the practical impact of management strategies regardless of their legal status, and create a more comparable dataset across different national contexts.

Cluster analysis was performed on the management strategies using hierarchical clustering through R project. Euclidean distance between countries was calculated using the “dist” function, and hierarchical clustering was conducted using Ward's method (ward.D2) with the “hclust” function from the “*stats*” package in R. The resulting dendrogram was visualized, and countries were grouped into four clusters using the “cutree” function. Linear regression analysis was performed to examine the relationship between the number of invasive species (dependent variable) and various management strategies (independent variable). This analysis used the “lm” function in R. To visualize the relationships between the dependent variable and each independent variable, box plots were created using the “boxplot” function.

## 3 Results

### 3.1 Basic characteristics of global IAPs lists

**Fig. 2:**
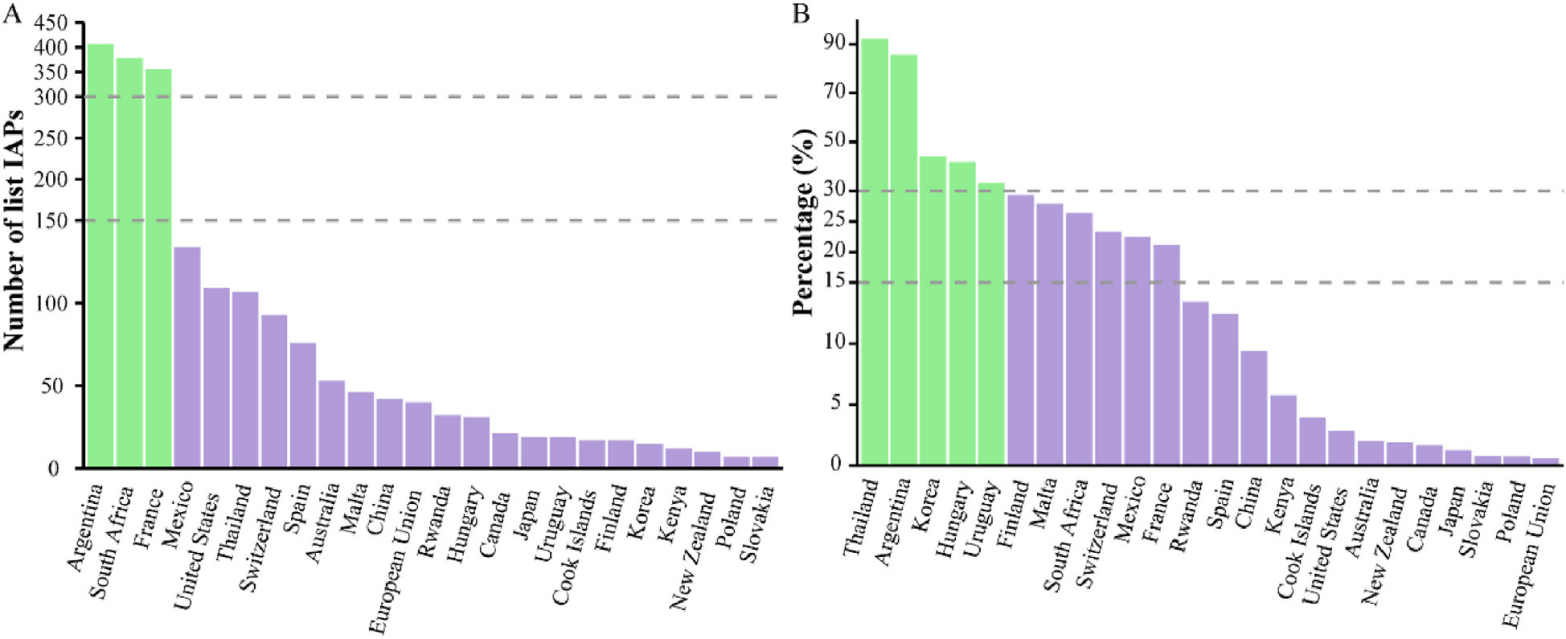
Invasive alien plants (IAPs) listed in national management inventories across countries. (A) Number of IAPs in each country's management list. Green bars indicate countries with over 150 listed IAPs. (B) Proportion of IAPs in each country's lists compared to the union of listed IAPs and the total number of introduced and invasive plants in GRIIS for each country. Green bars indicate countries where this proportion exceeds 30%. Gray dashed lines represent truncation thresholds. Countries are ordered by decreasing values in both panels.

The 24 countries which had national-level lists spanned Africa (3 countries), Asia (4 countries), Europe (8 countries and the EU), North America (3 countries), South America (2 countries), and Oceania (3 countries). France (363 species), South Africa (379 species), and Argentina (407 species) comprised the most extensive national catalogues of IAPs under management mandates (Fig. 2A; Tables S2 and S3). In contrast, Slovakia contained the shortest list with only 7 species. Compared to the total number of introduced and invasive plants in GRIIS for each country, only two countries, Thailand (92.24%) and Argentina (85.68%) had more than half species listed (Fig. 2B; Tables S2 and S3). Slovakia, Poland, and the EU had less than one percent of species listed (0.81%, 0.79%, and 0.62%, respectively) (Fig. 2B; Tables S2 and S3).

**Fig. 3:**
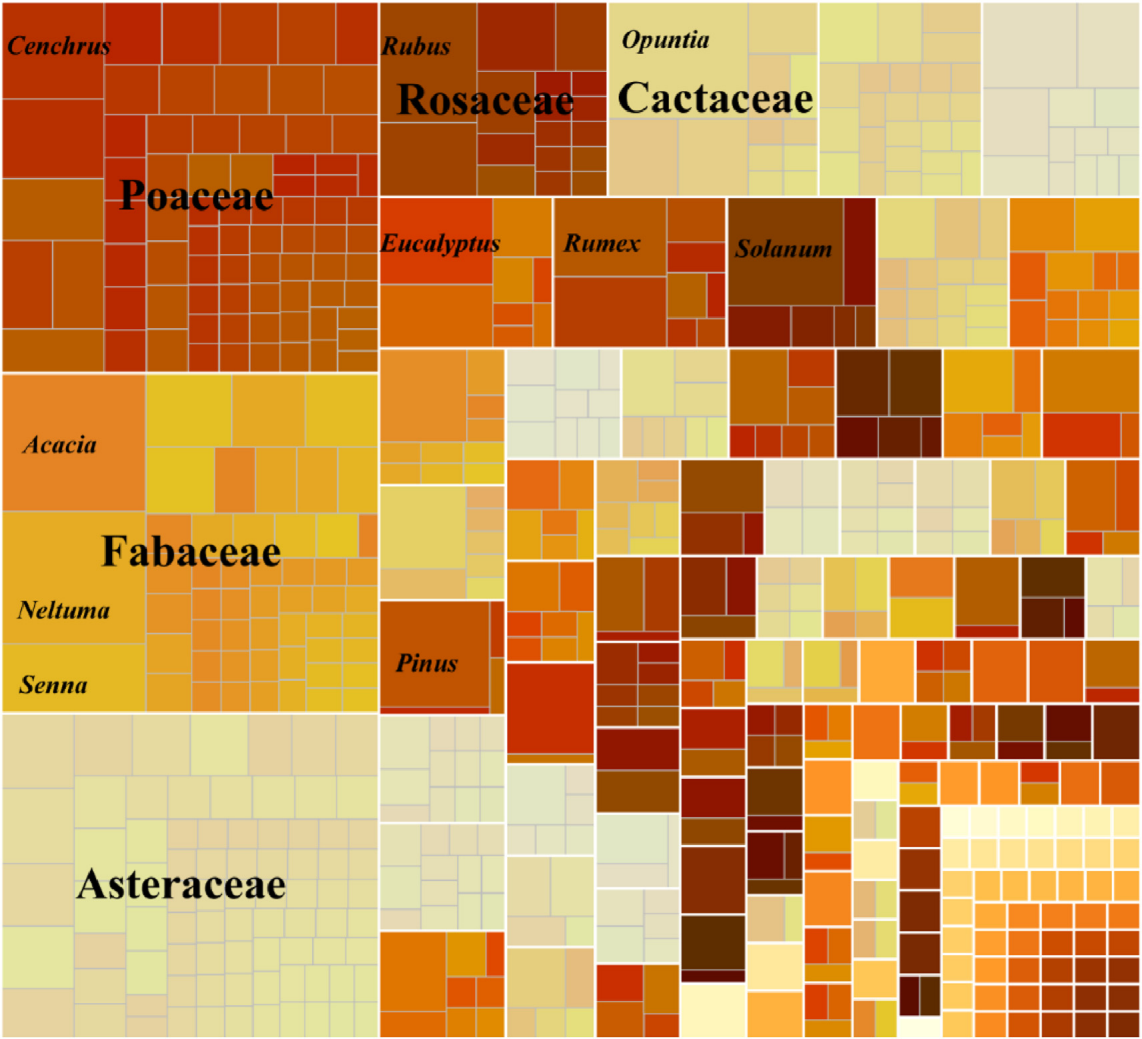
Treemap visualization of total invasive alien plants (IAPs) of management lists distribution across families and genera. The size of each rectangle represents the number of IAPs within a family or genus. The top 5 families and 10 genera are prominently displayed.

After standardization, the global IAPs lists comprised 1297 taxa, with the majority being species (Table S4). The lists also included 11 taxa identified only to the genus level (accounting for 0.85% of the total), 6 varieties (0.46%), 12 subspecies (1.00%), 1 form (0.08%), and 9 hybrids (0.69%). These 1297 taxa belong to 151 families and 676 genera, with 32 species of ferns, 22 species of gymnosperms, and 1243 species of angiosperms (Table S4). The five families with the highest number of species were Poaceae (154 species), Fabaceae (141 species), Asteraceae (135 species), Rosaceae (49 species), and Cactaceae (45 species) (Fig. 3). The ten genera with the highest number of species were *Acacia* (22 species), *Neltuma* (21 species), *Opuntia* (18 species), *Solanum* (14 species), *Pinus* (13 species), *Rubus* (13 species), *Cenchrus* (11 species), *Eucalyptus* (11 species), *Senna* (11 species), and *Rumex* (10 species) (Fig. 3). Fifty-two families and 430 genera were represented by only one species each (Fig. 3 and Table S4).

### 3.2 Listing patterns and main factors influencing the inclusion of IAPs in management lists

The distribution of families, genera, and species across national IAPs management lists revealed a pattern in which most species were listed by only a few countries, while a small number of species were widely recognized across multiple countries (Table 1). One thousand two hundred fifty-one species, 607 genera, and 102 families were listed in one to four countries; 45 species, 62 genera, and 34 families in five to nine countries; and 2 species, 7 genera, and 10 families in 10–14 countries (Table 1). No species or genera were listed in 15 or more countries, while three families appeared in 20 or more countries (Table 1). *Pistia stratiotes* and *Pontederia crassipes* were the most frequently listed species (11 countries each), *Solanum* and *Pontederia* were the most common genera (12 countries each), and Asteraceae and Fabaceae were the most widely recognized families (21 countries each) (Table S5).

**Table 1:** Frequency distribution of species, genera, and families in national invasive alien plants (IAPs) management lists.

No. of listing countries	No. of species	No. of genera	No. of families
≥ 20	0	0	3
15–19	0	0	2
10–14	2	7	10
5–9	45	62	34
1–4	1251	607	102

Six species, *Alternanthera philoxeroides*, *Cabomba caroliniana*, *Ludwigia grandiflora*, *Parthenium hysterophorus*, *Pontederia crassipes*, and *Ulex europaeus*, were included in the management lists across all six continents, while *Hydrocotyle ranunculoides*, *Lagarosiphon major*, *Microstegium vimineum*, *Myriophyllum aquaticum*, *Pistia stratiotes*, *Ricinus communis*, and *Salvinia molesta* were listed in five continents (Table S5). This widespread inclusion indicated a global consensus on the invasive status of these particular species.

**Fig. 4:**
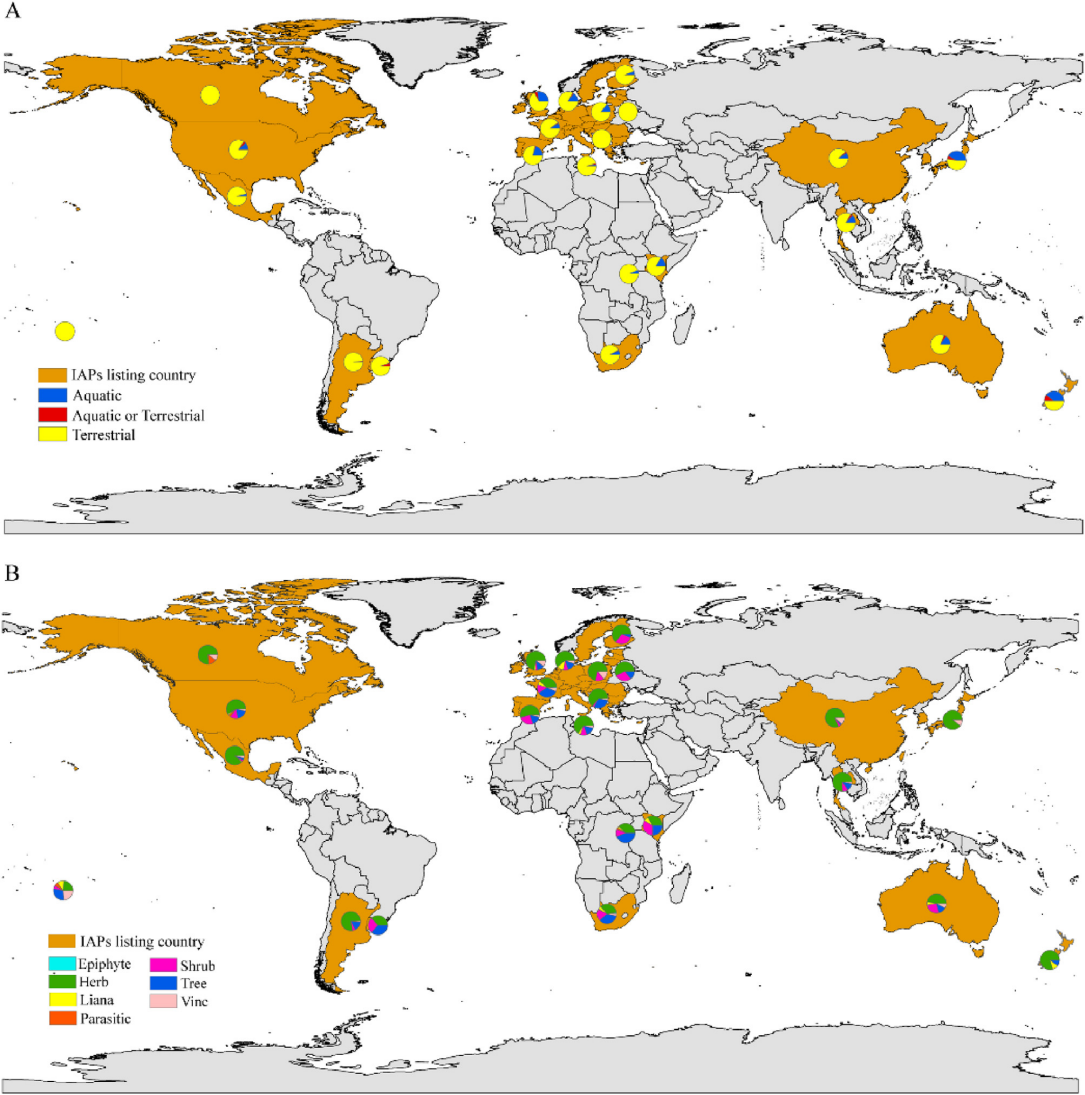
Global distribution of invasive alien plant (IAPs) by habitat types (A) and growth forms (B) across national management lists. Map lines delineate study areas and do not necessarily depict accepted national boundaries. Due to the inclusion of the European Union (EU) list, all EU countries are represented by orange-yellow coloring. The pie chart for the EU list is positioned over the United Kingdom for visualization purposes only.

Although there were few aquatic (67) and aquatic or terrestrial (23) IAPs, a significant proportion of countries (83.00%) had these species listed (Table 2 and Fig. 4A). The average distribution frequency was 2.57 for aquatic IAPs, and 1.65 for aquatic or terrestrial IAPs (Table 2). Terrestrial IAPs, with 1173 species, were found in all countries studied, with an average distribution frequency of 1.58 (Table 2). For growth forms of these IAPs, herbs were the most prevalent, with 738 IAPs listed in all countries studied and an average distribution frequency of 1.61 (Table 2 and Fig. 4B). Trees and shrubs followed, with 288 and 153 IAPs, respectively, both listed in 20 countries (Table 2 and Fig. 4B). However, shrubs had a higher average distribution frequency (1.82) compared to trees (1.55) (Table 2). Vines and lianas, each comprising 50 IAPs, were listed in 18 and 15 countries, with average distribution frequencies of 1.80 and 1.44, respectively (Table 2). Epiphytes and parasitic IAPs were the least represented growth forms, with 10 and 8 species listed in only two and six countries and average listing frequencies of 1.00 and 1.50, respectively (Table 2 and Fig. 4B).

**Table 2:** Distribution and frequency of invasive alien plants (IAPs) across different habitats and growth forms.

Habitats and growth forms	No. of species	No. of distributed countries	Average distribution frequency
Aquatic	67	19	2.57
Aquatic or terrestrial	23	15	1.65
Terrestrial	1207	24	1.57
Epiphyte	10	2	1.00
Herb	738	24	1.61
Liana	50	15	1.44
Parasitic	8	6	1.50
Shrub	153	20	1.82
Tree	288	20	1.56
Vine	50	18	1.80

Our analysis revealed significant differences in IAP listing frequencies across habitat types (*p* < 0.05) (Table S6 and Fig. S1). Aquatic habitats showed the highest listing frequency (2.57), followed by aquatic or terrestrial (1.65) and terrestrial habitats (1.57) (Table S6 and Fig. S1A). No significant differences were observed in listing frequencies among different growth forms (Table S6 and Fig. S1B). IAPs with more extensive native ranges showed lower occurrence frequencies (*p* < 0.05), indicating a negative correlation between native distribution range and inclusion in national management lists (Fig. S2).

### 3.3 Similarities, and intercontinental exchange among national lists

**Fig. 5:**
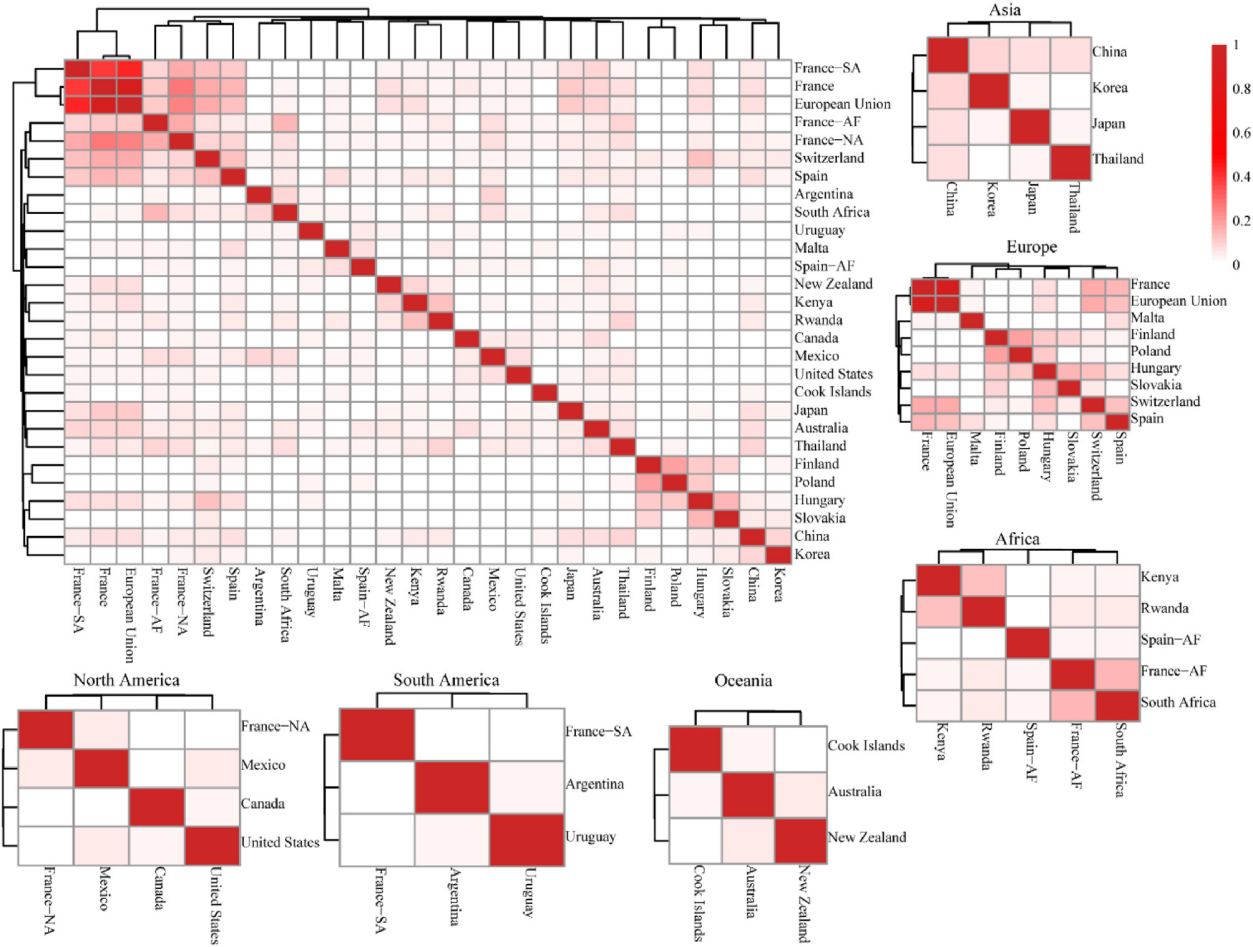
Similarities of invasive alien plant (IAPs) in the management lists among countries. NA = North America, SA = South America, AF = Africa. Notations combining a country name with a continent abbreviation represent that country's overseas territories in the respective continent.

The species composition of IAPs listed by the French mainland and the EU exhibited the highest similarity (0.91) (Fig. 5 and Table S7). Examining each continent separately, the average similarity coefficients were 0.06 for Asia, 0.05 for Africa, 0.08 for Europe, 0.05 for North America, 0.01 for South America, and 0.04 for Oceania (Fig. 5 and Table S7). However, the overall average similarity among countries and continents remained relatively low, with an average similarity of 0.04.

**Fig. 6:**
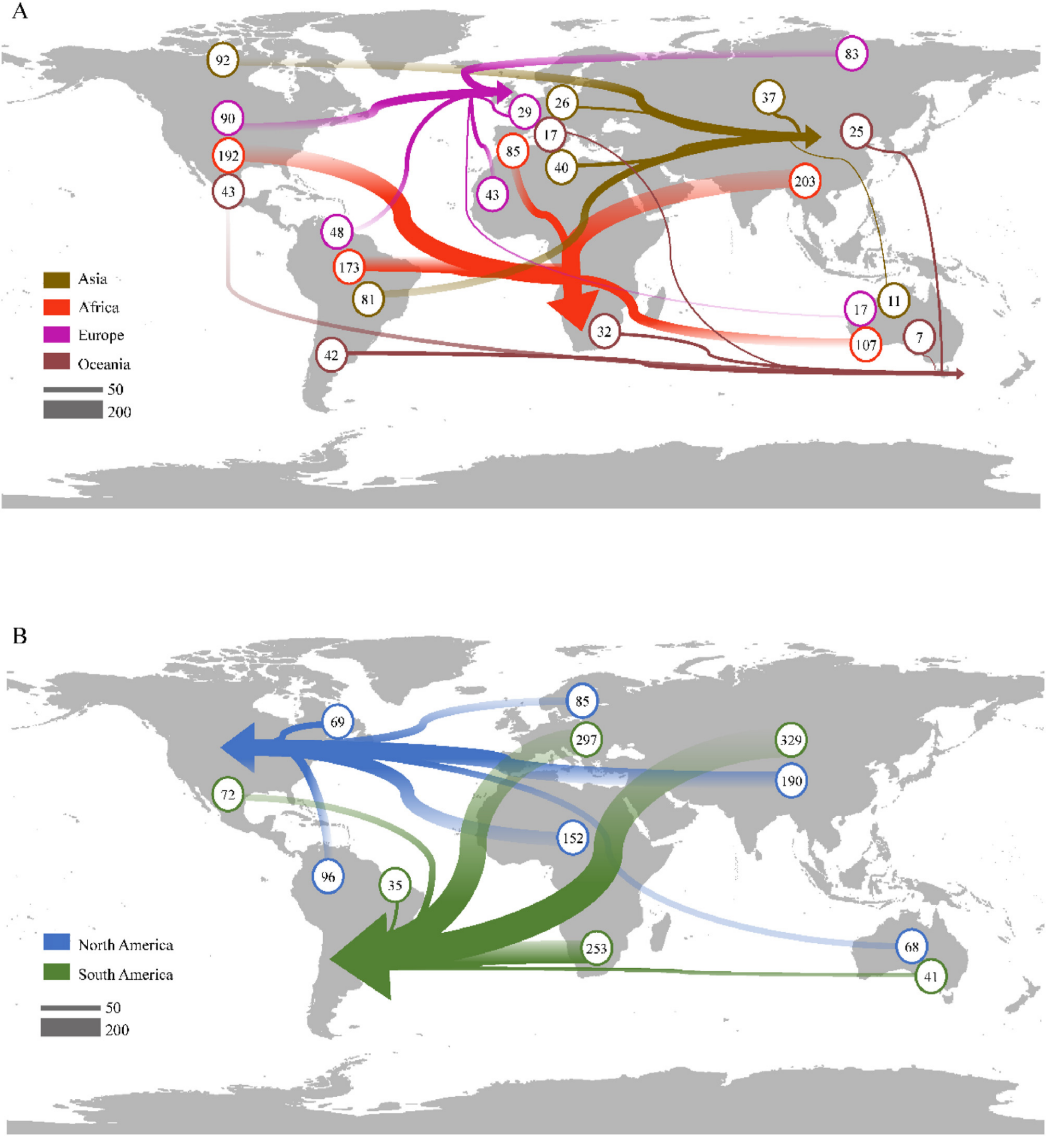
Intercontinental flows of invasive alien plants (IAPs) between native and invaded continents. (A) Origins of IAPs in Eastern Hemisphere continents (Asia, Africa, Europe, and Oceania). (B) Origins of IAPs in Western Hemisphere continents (North and South America). Line thickness represents the number of invasive species. Arrows point to invaded continents, with numbers indicating species count from each origin.

Native distributions of IAPs in management lists revealed distinctive patterns of intercontinental exchange (Fig. 6 and Table S8). For European countries, the majority of IAPs originated from North America (29.03%) and Asia (26.77%). Asian IAPs showed a significant number of species from North America (32.06%) and South America (28.22%). African IAPs mainly came from Asia (22.91%) and North America (21.67%). In North America, Asia (28.79%) and Africa (23.03%) were the primary sources of IAPs. South American IAPs mainly came from Asia (32.03%) and Europe (28.92%). Oceania's lists showed a more balanced distribution, with North America (25.90%) and South America (25.30%) as the main sources, followed by Africa (19.28%) and Asia (15.06%). Notably, Asia emerged as a significant contributor of IAPs in management lists across multiple continents, while South America and Oceania appeared to be less frequent sources in the lists of other regions (Fig. 6).

McNemar's test showed a significant directional exchange of IAPs among continents (χ^2^ = 40.71, df = 1, *p* = 1.766e^−10^). Further analysis revealed that most IAPs in the lists of the Eastern Hemisphere countries originated from the Western Hemisphere (581 species, 67.02%), while among the IAPs recorded in the Western Hemisphere, most species (382 species, 78.93%) originated from the Eastern Hemisphere. This result unveiled an interesting global pattern of IAPs exchange: there was a significant directional invasion between the Eastern and Western Hemispheres.

### 3.4 Characteristics of existing management measures in different countries

The number of IAS national management lists grew significantly over time: prior to 2015, only 11 lists existed, whereas this number more than doubled to 27 lists after 2015 (Fig. S3). While management lists inherently implied a degree of prioritization (Table 3), analysis of the list characteristics from 24 countries revealed that nine countries explicitly emphasized “priority” IAPs within their lists. This prioritization was manifested in two primary ways: clarifying the risk or impact level of species (Australia, Cook Islands, Malta, and Rwanda), or the management methods (Argentina, Japan, Cook Islands, Malta, Mexico, South Africa, Switzerland, and Thailand) (Table 3). Notably, Cook Islands and Malta employed both. Only Argentina, Switzerland, and Thailand's lists explicitly included potential IAPs. Ten countries' lists address priority areas. Malta, Mexico, and South Africa highlighted IAPs that threaten priority sites (protected Natural Areas, areas containing rare and/or endemic species, or islands). Cook Islands, Malta, New Zealand, Japan, France, and Spain were islands themselves or developed equivalent lists specifically for their dependent islands. Hungary and Kenya developed national-level management lists specifically for priority protected areas. The management measures associated with the lists showed that 15 countries had requirements to prevent or reduce the introduction, spread, and establishment of IAPs. Among these, 13 explicitly prohibited introduction into the country, 8 included natural ecosystem/environment management, and 11 addressed spread (including prohibited growing, selling, and moving) (Table 3). The documents associated with the lists from 13 countries explicitly mentioned control and eradication requirements, and from 16 countries were legal documents. Only 7 had clear penalties (Table 3).

**Table 3:** Framework analysis of list characteristics and management regulations across countries.

List/management	Mainly characteristic	Specific description	Countries
List characteristics	Divided levels	Risk/Impact assessment level	Australia, Cook Islands, Malta, Rwanda
		Management classification	Argentina, Cook Islands, Malta, Mexico, South Africa, Switzerland, Thailand
	Potential IAPs	Lists include potential invasive species	Argentina, Switzerland, Thailand
	Priority site IAPs	List highlights IAPs that threaten priority sites	Malta, Mexico, South Africa
		The list itself is developed for priority sites, such as islands	Cook Islands, France, Hungary, Japan, Kenya, Malta, New Zealand, Spain
Management regulations	Preventing or reducing the introduction and establishment	Forbidden introduction into the country (Country ban)	Argentina, Canada, European Union, Finland, France, Japan, Korea, Mexico, New Zealand, Slovakia, South Africa, Spain, United States
		Include natural ecosystem/environment management (Nature ban)	European Union, Finland, France, Hungary, Kenya, Korea, Mexico, Spain
		Prohibited spreading (Spread ban, including prohibited growing, selling, moving)	Canada, European Union, Finland, France, Hungary, Japan, Korea, Slovakia, South Africa, Spain, United States
	Eradicating or controlling	Strategies for managing established IAPs (Control)	Argentina, China, Cook Islands, European Union, Finland, Hungary, Japan, Korea, Poland, Slovakia, South Africa, Spain, United States
	Legal support	Legal documents	Argentina, Canada, European Union, Finland, France, Hungary, Japan, Kenya, Korea, Mexico, New Zealand, Poland, Slovakia, South Africa, Spain, United States
		Legal penalties (Penalty)	European Union, Finland, Japan, Kenya, Korea, Slovakia, Spain

Examining the correlation between list characteristics and management approaches revealed that countries with legal support in their management strategies were less likely to further categorize species into priority levels (Fig. S4). Only three countries (Argentina, Mexico, and South Africa) were exceptions to this trend, all reflecting species prioritization in their management approaches. Notably, ten countries demonstrated a comprehensive management policy by combining “Legal Support” with two additional management requirements. This indicated a more robust and well-rounded approach to invasive species management in these nations. Furthermore, 75.00% of countries with official lists had implemented further species prioritization within their inventories.

**Fig. 7:**
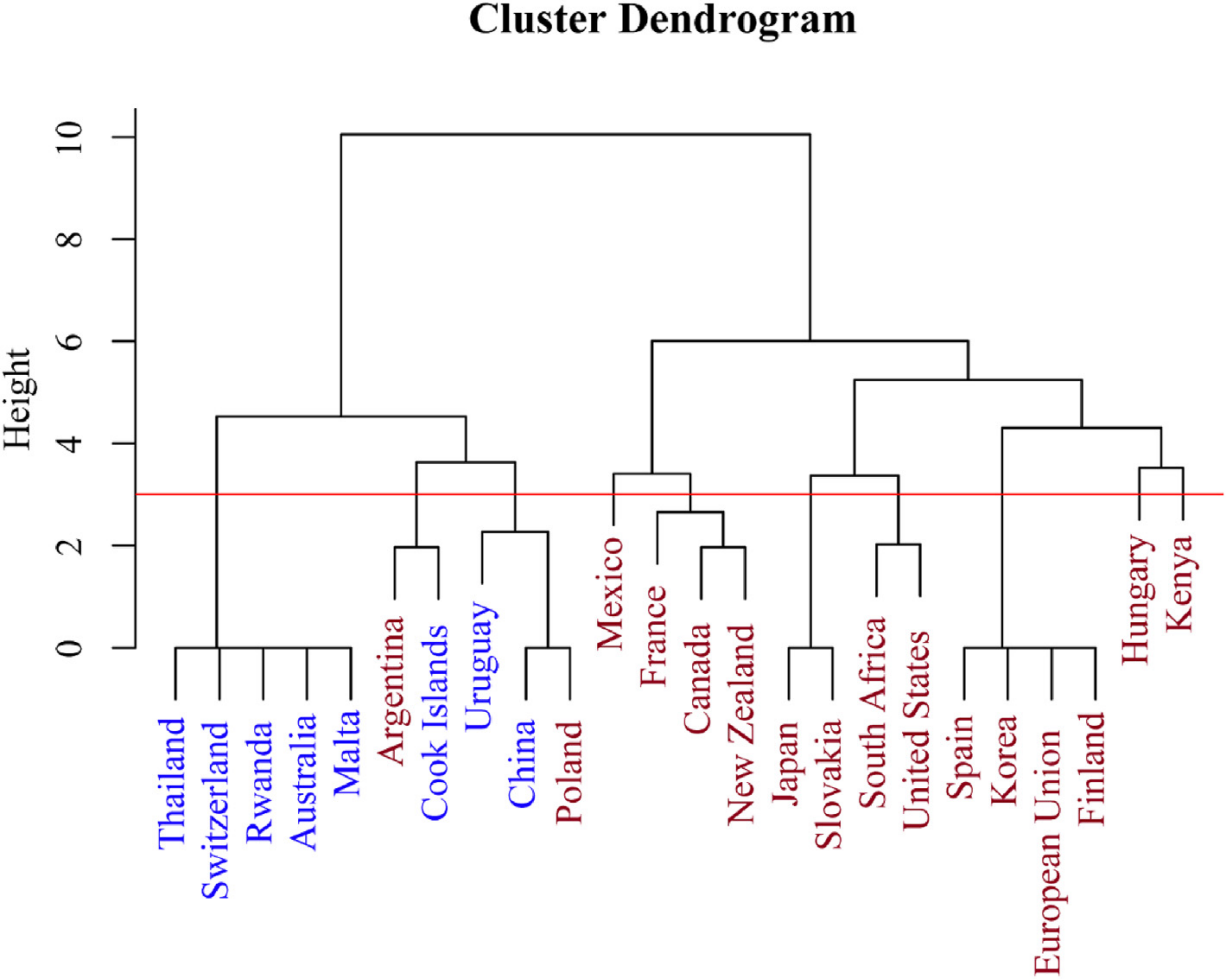
Cluster dendrogram analysis based on management approaches of invasive alien plants (IAPs) lists across countries. Blue text indicates countries' IAPs lists are official lists, while red text countries' IAPs lists are legal documents.

The cluster dendrogram analysis based on the management approach revealed two main clusters (Fig. 7). One cluster consisted primarily of office lists, with the exceptions of Argentina and Poland. The other cluster was comprised entirely of countries using legal documents. This clear separation suggested that name lists that carried legal authority differed substantially in their management compared to those that merely served as office lists. The results indicated that the legal status of these name lists was a key factor influencing the administrative practices surrounding them.

The analysis of management measures associated with IAS lists revealed that the implementation of invasion level and introduction prohibition was significantly positively associated with the number of listed species (*p* = 0.0460，*p* = 0.0295), while the presence of penalties was significantly negatively correlated (*p* = 0.0275) (Fig. S5). These findings suggested that countries’ preferences in measure selection were influenced by their specific conditions and factors.

### 3.5 Towards a new generation of IAS lists: tiered classification for achieving Target 6

**Fig. 8:**
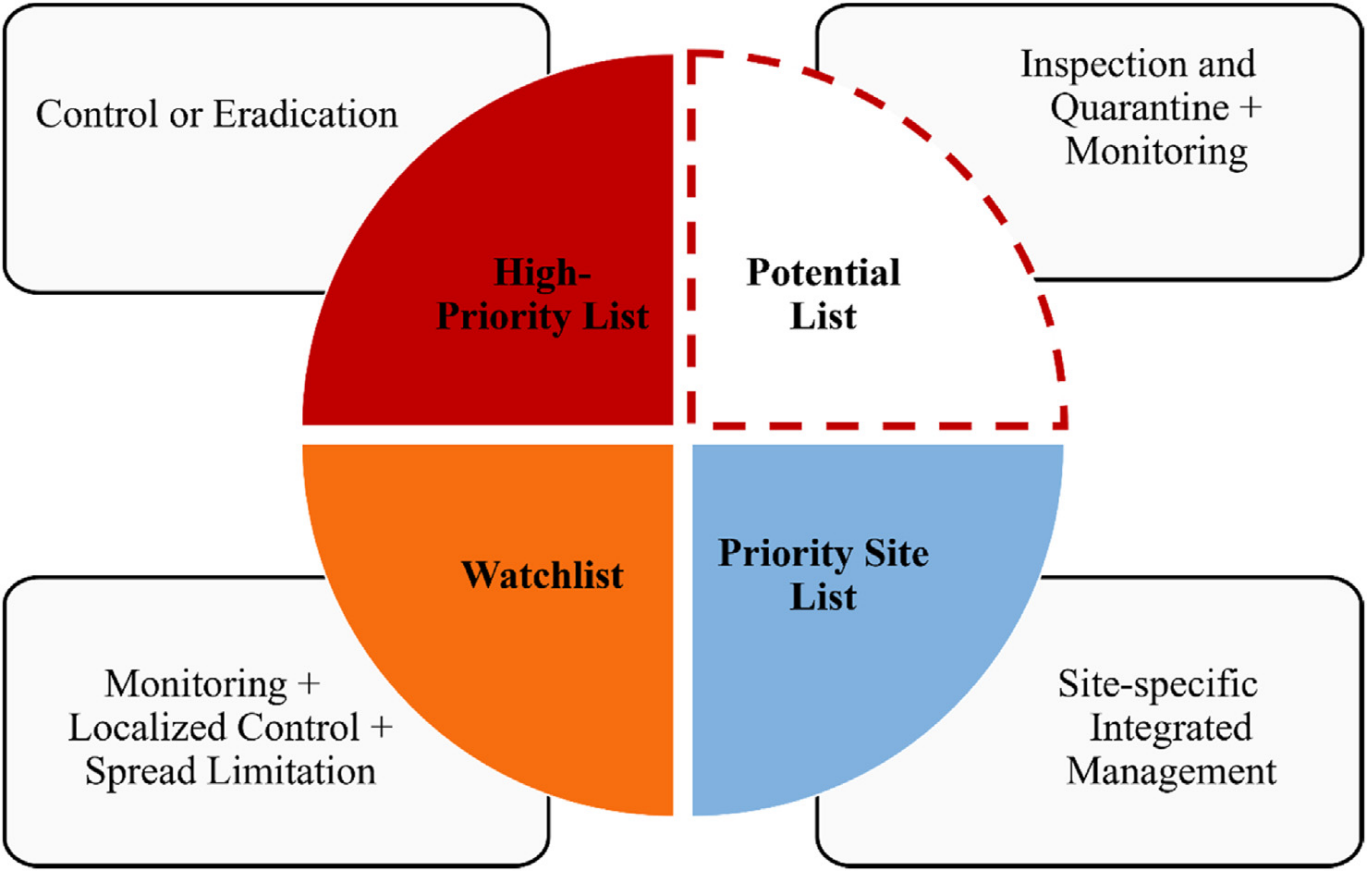
A new era in tiered classification systems for invasive alien species (IAS) management: Towards achieving Target 6.

To effectively meet the challenges posed by Target 6, a new generation of IAS lists based on a tiered classification system is proposed in this study (Fig. 8). This comprehensive approach encompasses four distinct categories: (1) High-Priority List, primarily including IAS that have caused significant ecological or economic damage, with management focusing on control and eradication, aiming to reduce existing impacts and prevent further damage. (2) Watchlist, species in early invasion stages or causing limited ecological disturbances, requiring a management approach combining vigilant surveillance, targeted intervention in specific areas, and measures to curtail further dissemination. The primary objectives are to impede additional proliferation and evaluate these species’ potential long-term consequences on native ecosystems and biodiversity. (3) Potential List, comprising species with invasive characteristics in other regions or intentionally introduced horticultural species with proliferation potential, emphasizing rigorous inspection, quarantine, and monitoring protocols, aiming to establish early warning mechanisms and implement interception strategies; (4) Priority Site List, addressing invasive species in ecologically sensitive regions and high-value conservation zones, employing site-specific integrated approaches, aiming to safeguard critical ecosystems and biodiversity hotspots, preserving their ecological integrity and the unique assemblages of species they support.

## 4 Discussion

Global efforts to mitigate the threats posed by IAS have led to diverse management strategies and policies. However, more research is needed focusing on national IAS management lists, which are critical tools for identifying and prioritizing species for control. The concept of “priority species” was proposed in the Aichi Targets, but the interpretation and application of this concept vary among countries. This study addressed these knowledge gaps by analyzing the composition, characteristics, and management measures associated with national IAPs lists from 24 countries worldwide. The findings revealed significant commonalities and disparities among these lists, reflecting the inherent invasiveness of certain species and the differing approaches to IAS prioritization across nations. This study also explored the underlying factors shaping national lists, the alignment of current management practices with global targets, and the implications for strengthening international collaboration in IAS management.

### 4.1 Similarities and differences among national lists

Certain IAPs were widely recognized for their extensive harm. For example, *Pistia stratiotes* and *Pontederia crassipes* were included in the management lists of 11 countries (45.8%). *Pistia stratiotes,* an ancient species with native ranges in both the Neotropics and Afrotropics (Murphy et al., 2019), was documented as introduced in 107 countries or islands according to the Global Biodiversity Information Facility (GBIF, https://www.gbif.org/species/2870583, consulted in March 2024). Similarly, *Pontederia crassipes*, native to South Tropical America, was recorded as introduced in 147 countries or islands (GBIF, https://www.gbif.org/species/2765940, consulted in March 2024). The global invasiveness of *Alternanthera philoxeroides*, *Cabomba caroliniana*, *Ludwigia grandiflora*, *Parthenium hysterophorus*, *Pontederia crassipes*, and *Ulex europaeus* was further evidenced by their inclusion in the management lists across six continents. Notably, among these seven widely distributed IAPs, four are aquatic invaders (57.1%). Although terrestrial herbaceous plants constitute the majority of IAPs, aquatic IAPs were more frequently listed in the management lists. The heightened attention to aquatic IAPs in management strategies could be attributed to the severity of their impacts and the challenges associated with their control. Aquatic ecosystems are particularly vulnerable to biological invasions (Havel et al., 2015), and the introduction of aquatic IAPs can not only alter the community composition of these ecosystems (Li et al., 2024; Madsen et al., 1991) but also impede river flow (Thouvenot et al., 2013), channel (Bunn et al., 1998), and related aquatic economic activities (Halstead et al., 2003; Hussner et al., 2017; Li et al., 2022). Macêdo et al. (2024) assessed the global economic impact of aquatic IAPs and found that between 1975 and 2020, aquatic and semi-aquatic IAPs caused a total cost exceeding $32 billion to the global economy, with $1.6 billion for management.

Our study revealed a significant negative correlation between the number of continents in an IAP's native range and its frequency of inclusion in management lists (*p* < 0.05). This finding stands in contrast to previous predictive studies on plant invasiveness, which hypothesized that species with wider native ranges might have higher invasive potential (Cadotte et al., 2006; Pyšek et al., 2009) due to two main factors: (1) species with wider native ranges are more likely to be transported by humans to other regions (Forcella and Wood, 1984; Pyšek et al., 2009); (2) the traits that determine a species' native range also determine its range in the invaded area (Thompson et al., 1995; Pyšek et al., 2009). This discrepancy suggested a potential bias between management lists and invasiveness risk assessments. IAPs with limited native ranges, due to their stronger “alien” nature, might be more readily included in management lists of countries, while widespread IAPs might be considered “native” in many countries and consequently excluded from management lists.

Despite some consensus in management lists across countries, significant variations persisted, primarily in species quantity and composition. Our study revealed that the number of listed IAPs correlated significantly with the presence of level differentiation (Divided Levels), country entry prevention (Country Ban), and clear penalties (Penalties). This indicated that countries consider factors such as impact severity, management complexity, and legal frameworks when formulating their management lists. Countries that adopt “Divided Levels” and “Country Ban” strategies exhibited more comprehensive lists, likely their initial design for tiered management and diverse approaches, rather than focusing solely on high-priority IAS. Additionally, jurisdictions with stringent introduction, importation, or trade regulations often included potential IAPs in their lists. On the other hand, management lists with clearer penalty measures typically contained fewer species, indicating a more cautious approach to species inclusion. This strategy may aim to avoid excessive impact on socio–economic activities or triggering controversies by focusing on high-risk species rather than adopting a broad management list. Notably, we found no significant correlation between management measures and the proportion of IAPs in the management lists relative to the total number of introduced and invasive plants in GRIIS. This might be due to the lack of a unified global standard for the proportion of species to be included in management lists.

European countries show a higher resemblance compared to other regions. Especially, France and the EU demonstrated the highest similarity (0.91) in their IAP lists. This elevated congruence could be primarily attributed to the EU's coordinated efforts in invasive species management, particularly through the EU Regulation on Invasive Alien Species (1143/2014). Moreover, France's unique position as the largest EU member state by area, encompassing diverse ecosystems, likely contributes significantly to this similarity by capturing a substantial subset of species of EU-wide concern. Despite this notable case of high similarity, the general landscape of IAP listing across countries remains diverse. National lists were typically derived from risk assessments, there existed a diverse array of risk assessment systems (Roy et al., 2018), with countries often employing their own frameworks. Moreover, some lists were generated through expert assessments and stakeholder assessments, such as the National Priority List of Exotic Environmental Pests, Weeds and Diseases (EEPL) of Australia (https://www.agriculture.gov.au/biosecurity-trade/policy/risk-analysis/weeds/system, consulted in September 2023). However, these approaches may be constrained by the knowledge limitations of the experts and stakeholders involved, leading to substantial differences even between neighboring countries. In addition, IAPs lists from different countries were published by various departments, resulting in divergent species focus. The lists analyzed in this study originate from departments spanning multiple sectors, such as ecological environment, agriculture, forestry, fisheries, and food. In some countries, the lists were jointly published by multiple departments (China and France). On the other hand, in some countries, the lists were directly published by the national government bodies. This departmental diversity influences the types of IAPs prioritized. For instance, lists published by agricultural departments may emphasize species impacting agricultural production, while those from environmental departments may adopt a more comprehensive approach, considering impacts on natural ecosystems.

### 4.2 Geographical patterns in IAPs donor and recipient: insights from management lists

While selecting IAPs for management lists involved complex considerations, including management practicalities, these lists might to some extent reflect the IAPs that countries considered most threatening or in need of management. The native origins of IAPs in each continent's management lists revealed that species native to Asia constituted a significant proportion of IAPs of concern across multiple continents. Notably, despite our focus on management-listed species, which is a relatively limited dataset and potentially represents only the most problematic subset of IAPs, this finding aligned with previous global naturalization studies, which observed that the flow of naturalized plant species indicated temperate Asia as one of the major donors (van Kleunen et al., 2015). This consistency indirectly reflected the strong adaptability of species originating from Asia in other continents, and emphasizes that Asian species are not only significant in numbers but also receive special attention in management priorities. Darwin hypothesized that due to their more competitive evolutionary history, Northern Hemisphere species are inherently more competitive than Southern Hemisphere species (Darwin, 1859), facilitating their successful naturalization (van Kleunen et al., 2015). Our analysis corroborated this hypothesis, revealing fewer IAPs originating from Southern Hemisphere continents, specifically South America and Oceania. The minimal contribution of IAPs native to Oceania to IAPs in management lists of other continents might be attributed to Australia's prolonged biogeographical isolation and arid climate, which have resulted in a phylogenetically distinct native flora correct to (Crisp et al., 2004; Byrne et al., 2008). This distinctiveness potentially limited the adaptability of Australian flora in ecosystems of other continents, such as Europe (van Kleunen et al., 2015).

The IAPs in these management lists exhibited a significant directional invasion pattern between the Eastern and Western Hemispheres, consistent with the Old World versus the New World model of biological invasions (Castri, 1989; Lonsdale, 1999). While historical anthropogenic factors such as trade routes and colonial expansion influenced species exchange between the Old and New Worlds (Hulme, 2009), the persistence of this pattern in contemporary management lists, despite intensified globalization in recent decades, suggested that it may reflect ecological and evolutional processes rather than merely anthropogenic influences. Long-term isolation between hemispheres resulted in divergent evolutionary trajectories and unique phylogenetic associations within each region (Qian and Ricklefs, 2004). Consequently, introductions of inter-hemispheric species often represented novel phylogenetic elements in recipient communities, potentially conferring advantages to introduced species through multiple mechanisms (Strauss et al., 2006). These advantages included the absence of co-evolved natural enemies (Keane and Crawley, 2002; Mitchell and Power, 2003), possession of novel functional traits facilitating niche occupation (Brym et al., 2011; Mathakutha et al., 2019), and the lack of effective defensive strategies in native species (Callaway and Ridenour, 2004).

### 4.3 Gaps in achieving Target 6 and addressing implementation challenges

The prioritization of IAS for cost-effective management was emphasized in Aichi Target 9, aiming to identify and document “priority” IAS (Krug et al., 2009; McGeoch et al., 2016). However, our analysis of national management lists revealed a relative weakness in species prioritization, with only 37.5% of examined countries explicitly indicating high-risk or high-priority management species. This lack of explicit prioritization might stem from several factors, including insufficient baseline data, impacting the accuracy of risk assessments and prioritization processes (McGeoch et al., 2016; Essl et al., 2018). Despite data limitations, effective prioritization could be achieved by combining expert knowledge with evidence from other regions (McGeoch et al., 2016). Species causing severe impacts were often widely recognized, even in countries lacking detailed invasion threat information (Hayes and Barry, 2008). Furthermore, the Environmental Impact Classification of Alien Taxa (EICAT) assessment tool also considered the maximum historical or current impact recorded for a species anywhere as a crucial evaluation criterion (Blackburn et al., 2014; Hawkins et al., 2015; McGeoch et al., 2016). Therefore, based on the 38 lists from 24 countries, the species consistently recognized as problematic across these lists can serve as a valuable reference for worldwide countries in identifying their priority IAPs. For instance, our analysis revealed the five most frequently listed and widely invasive harmful species, *Pistia stratiotes*, *Pontederia crassipes*, *Salvinia molesta*, *Cabomba caroliniana*, and *Ulex europaeus*, which were listed by at least eight countries (33.3% of the analyzed) and invaded into five or more continents. Interestingly, among these five species, *Pistia stratiotes* and *Cabomba caroliniana* were not included in the “100 of the World's Worst Invasive Alien Species” list (Lowe et al., 2000), highlighting potential discrepancies between national management priorities and global assessments of IAS impacts.

The increasing focus on potential IAS represented a significant shift in invasion management strategies (Seebens et al., 2017). Effective prevention and control of potential invaders at the earliest stages could substantially reduce subsequent management costs (Holden et al., 2016; Epanchin-Niell, 2017; Diagne et al., 2021). This evolving understanding was reflected in the Kunming-Montreal Global Biodiversity Framework's Target 6, which explicitly addresses potential IAS for the first time, a consideration absent from the previous Aichi Target 9. However, our analysis of global management lists revealed a considerable gap between this new requirement and current practices, with only three countries including potential IAPs in their lists. Researchers have developed and proposed various approaches to enhance the identification of potential IAS (Peterson, 2003; Roy et al., 2014). One relatively straightforward method was to learn from the “experience” of neighboring countries, leveraging existing knowledge and data from geographically proximate regions to inform risk assessments (McGeoch et al., 2016; García-Díaz et al., 2018). Natural dispersal from adjacent countries or regions was recognized as one of the major pathways for IAPs invasion (Wilson et al., 2009), exemplified by the invasion of *Ageratina adenophora* in China naturally dispersed from a neighboring country (Xu et al., 2006). Therefore, the inconsistency between neighboring countries' lists suggested that species listed as invasive in one country could serve as indicators of potential invaders in adjacent regions. Furthermore, our identified patterns of species exchange between the Eastern and Western Hemispheres provided a framework for predicting potential invasions. This inter-hemispheric species pattern could serve as a reference for risk assessment in plant trade and introduction, emphasizing the need to consider potential invasive risks when evaluating plant exchanges between these hemispheres.

Target 6 introduced a novel emphasis on the eradication or control of IAS in priority sites, particularly islands, reflecting the growing focus in invasion ecology on managing specific high-value or high-risk areas (Dawson et al., 2017; Russell and Kueffer, 2019). Clearly defining which areas are priority sites is crucial for achieving this goal. The priority of islands in IAS management was repeatedly emphasized by many studies due to the vulnerability of island ecosystems and the high proportion of endemic and endangered species, which made them more sensitive to the impacts of IAS (Bellard et al., 2013, 2016; Pyšek et al., 2020). In addition, areas with important biodiversity conservation value, such as intact forests, grasslands and wetlands, as well as nature reserves, should be considered as high-priority sites for IAS management due to their critical importance for ecosystem services (Gallardo et al., 2017; Potgieter et al., 2022). However, apart from island nations, only seven countries incorporated priority sites or overseas island territories in their lists. Therefore, to meet the requirements of Target 6, it is necessary to first clarify the current status of IAS in key protected areas of domestic natural ecosystems or affiliated islands and establish targeted lists. These lists form the foundation for achieving the eradication or control of IAS in priority areas.

Preventing or reducing the introduction and establishment, as well as eradicating or controlling invasive species, are two important actions emphasized by Target 6. Given the presence of clear lists, implementing relevant actions is critical to achieving management goals. The diversity in the formulation contexts of these lists corresponds to heterogeneous action strategies, with particularly pronounced disparities observed between legally binding lists and official lists, manifesting distinct patterns in their associated management interventions. Legally binding lists predominantly mandated measures for preventing or reducing the introduction and establishment of IAS, with Poland being the sole exception due to its policy's direct focus on control and eradication. In contrast, official lists generally lacked explicit requirements for such preventive actions. The prevention of entry and spread of IAS was the predominant strategy among nations, with eight countries explicitly mandating measures against IAS introduction or proliferation in natural environments. Notably, five countries with legal frameworks omitted requirements for “Control and Eradication”. Furthermore, despite the prevalence of legally binding lists, only seven nations delineated explicit punitive measures. This policy landscape demonstrated an apparent predilection for preventive strategies in IAS management. The preference for prevention aligned with its superior cost-effectiveness and ease of implementation compared to control and eradication (Leung et al., 2002; Hulme, 2009). However, for established invasions, control or eradication measures become imperative, albeit more complex in execution (Hulme, 2006). Historical data indicated that the efficacy of these measures was contingent upon the characteristics of invaded areas (Myers et al., 2000; Hulme, 2006). Moreover, inadequate risk assessment in controlling established IAS populations could precipitate unforeseen adverse outcomes (Kopf et al., 2017). Consequently, the application of control or eradication measures necessitated comprehensive survey data, rigorous regulatory frameworks, and meticulous risk assessment and prediction protocols.

### 4.4 Challenges and adaptive strategies for implementing tiered IAS classification lists

The new IAS classification system enables targeted resource allocation across invasion stages and impact levels, enhancing prevention, control, and ecosystem protection through differentiated management strategies. Its adaptability to emerging threats supports the achievement of 10.13039/100004791Target 6's objectives in managing IAS impacts on biodiversity and ecosystem services. However, the effective implementation of this system requires careful navigation of several aspects. First, data intricacies and taxonomic challenges require careful attention. The accurate classification of species as native or alien is fundamental yet complex in invasion biology, with challenges including limited historical data, scale-dependent criteria, and complex scenarios (Latombe et al., 2017; Essl et al., 2018). Furthermore, in our study, 11 countries adopted the “spp.” form for 30 genus-level taxa, reflecting the challenges of handling species complexes in species lists, which enhances biosecurity but increases compliance complexity (García-de-Lomas and Vilà, 2015). This approach highlights the limitations of relying solely on the biological species concept in invasion management. Secondly, resource allocation across different tiers of the IAS management system presents significant challenges, balancing immediate control measures for high-priority species with vigilant monitoring of potential threats. The increasing number of managed species not only complicates regulatory compliance for various stakeholders but also necessitates striking a delicate balance between comprehensive coverage and manageable implementation for the system's effectiveness and sustainability. Lastly, establishing a list of potential invasive species involves the challenge of predicting future impacts based on limited data. Furthermore, the inclusion of these species in management lists may encounter resistance from various parties that benefit from their presence (Dehnen-Schmutz, 2011).

To address these challenges, we recommend adopting global shared databases such as GIATAR for accurate species assessment and classification, which offers comprehensive, up-to-date information on invasive species distribution and traits, facilitating more precise risk assessments and informed management decisions (Saffer et al., 2024). We propose following the approach suggested by Pyšek et al. (2013). They recommend using the lowest possible taxonomic resolution while clearly indicating any uncertainty. Furthermore, better integration of classical alpha taxonomy and modern genetic taxonomic approaches can improve species identification accuracy and refine taxonomic classification at the level of populations and genotypes (Pyšek et al., 2013). Optimizing resource allocation is crucial, with a focus on prioritizing high-impact species and critical ecosystems, while implementing early monitoring for watchlist species and periodic evaluations of potential invaders (McGeoch et al., 2016). For resource-limited countries, a phased implementation approach is advisable, initially concentrating efforts on high-impact species and key ecosystems. Collaboration stands as a cornerstone of effective IAS management, with international cooperation and multi-stakeholder partnerships proving instrumental in maximizing the impact of control strategies (Packer et al., 2017). These collaborative frameworks, involving government agencies, academic institutions, NGOs, and local communities, facilitate comprehensive knowledge exchange, optimize resource allocation, and enable coordinated actions across diverse sectors and geographical boundaries, significantly enhancing management outcomes (Novoa et al., 2018). The engagement of diverse stakeholders ensures that management strategies are not only scientifically sound but also socially acceptable and practically implementable (Shackleton et al., 2019).

## 5 Conclusions

The management list of IAS usually encompassed the most prioritized species for management and action, directly influencing the achievement of Target 6 goals in the Kunming-Montreal Global Biodiversity Framework. Based on the analysis of IAS management lists from 24 countries, our study identified globally recognized harmful IAPs and revealed key patterns: the prevalence of aquatic plants in management lists, the dominance of species from Asia as major donors of IAPs, and a clear directional invasion pattern between the Eastern and Western Hemispheres. Furthermore, we evaluated the gaps in achieving Target 6 from the list characteristics of the management strategies associated with these lists. To address these challenges, we propose a tiered classification system for IAS lists, encompassing High-Priority, Watchlist, Potential, and Priority Site categories, which aims to enhance the effectiveness of IAS management by tailoring strategies to different invasion stages and ecological contexts. This tiered classification list may offer a flexible approach that is adaptable to emerging threats and changing environmental conditions. Challenges remain in implementing the tiered classification list approach. Future research should include establishing criteria for determining species numbers in each tier, developing standardized risk and impact assessment protocols, and expanding the analysis to include more countries and taxonomic groups. Evaluating the effectiveness of the tiered classification system through field trials and long-term monitoring is also necessary, as is exploring the integration of emerging technologies in IAS detection and management.

## CRediT authorship contribution statement

**Fei-Fei Li:** Writing – review & editing, Writing – original draft, Visualization, Formal analysis, Data curation, Conceptualization. **Qiang Hao:** Writing – review & editing, Data curation. **Xia Cui:** Writing – original draft, Data curation. **Ruo-Zhu Lin:** Writing – review & editing, Writing – original draft, Project administration, Formal analysis. **Bin-Sheng Luo:** Writing – review & editing, Visualization. **Jin-Shuang Ma:** Writing – review & editing, Writing – original draft, Project administration, Data curation, Conceptualization.

## Declaration of competing interest

The authors declare that they have no known competing financial interests or personal relationships that could have appeared to influence the work reported in this paper.

## References

[bib1] Bates D., Mächler M., Bolker B. (2014).

[bib2] Bellard C., Thuiller W., Leroy B. (2013). Will climate change promote future invasions?. Glob. Change Biol..

[bib3] Bellard C., Cassey P., Blackburn T.M. (2016). Alien species as a driver of recent extinctions. Biol. Lett..

[bib4] Bertolino S., Sciandra C., Bosso L. (2020). Spatially explicit models as tools for implementing effective management strategies for invasive alien mammals. Mamm. Rev..

[bib5] Blackburn T.M., Essl F., Evans T. (2014). A unified classification of alien species based on the magnitude of their environmental impacts. PLoS Biology.

[bib6] Booy O., Robertson P.A., Moore N. (2020). Using structured eradication feasibility assessment to prioritize the management of new and emerging invasive alien species in Europe. Glob. Change Biol..

[bib7] Brasier C. (2008). The biosecurity threat to the UK and global environment from international trade in plants. Plant Pathol..

[bib8] Brym Z.T., Lake J.K., Allen D. (2011). Plant functional traits suggest novel ecological strategy for an invasive shrub in an understorey woody plant community. J. Appl. Ecol..

[bib9] Bunn S., Davies P., Kellaway D. (1998). Influence of invasive macrophytes on channel morphology and hydrology in an open tropical lowland stream, and potential control by riparian shading. Freshw. Biol..

[bib10] Byrne M., Yeates D., Joseph L. (2008). Birth of a biome: insights into the assembly and maintenance of the Australian arid zone biota. Mol. Ecol..

[bib11] Cadotte M.W., Murray B.R., Lovett-Doust J. (2006). Ecological patterns and biological invasions: using regional species inventories in macroecology. Biol. Invasions.

[bib12] Callaway R.M., Ridenour W.M. (2004). Novel weapons: invasive success and the evolution of increased competitive ability. Front. Ecol. Environ..

[bib13] Cameron E.K., Vilà M., Cabeza M. (2016). Global meta-analysis of the impacts of terrestrial invertebrate invaders on species, communities and ecosystems. Global Ecol. Biogeogr..

[bib14] Capinha C., Essl F., Seebens H. (2015). The dispersal of alien species redefines biogeography in the Anthropocene. Science.

[bib15] Castri D., Drake J.A., Mooney H.A., di Castri F. (1989). Biological Invasions: A Global Perspective.

[bib16] Crisp M., Cook L., Steane D. (2004). Radiation of the Australian flora: what can comparisons of molecular phylogenies across multiple taxa tell us about the evolution of diversity in present–day communities?. Philos. Trans. R. Soc. Lond. B-Biol. Sci..

[bib17] Cuthbert R.N., Diagne C., Haubrock P.J. (2022). Are the “100 of the world's worst” invasive species also the costliest?. Biol. Invasions.

[bib18] Darwin C. (1859).

[bib19] Dawson W., Moser D., Van Kleunen M. (2017). Global hotspots and correlates of alien species richness across taxonomic groups. Nat. Ecol. Evol..

[bib20] Dehnen-Schmutz K. (2011). Determining non-invasiveness in ornamental plants to build green lists. J. Appl. Ecol..

[bib21] Diagne C., Leroy B., Vaissière A.-C. (2021). High and rising economic costs of biological invasions worldwide. Nature.

[bib22] Elton C.S. (1958).

[bib23] Engemann K., Sandel B., Boyle B. (2016). A plant growth form dataset for the New World. Ecology.

[bib24] Epanchin-Niell R.S. (2017). Economics of invasive species policy and management. Biol. Invasions.

[bib25] Essl F., Bacher S., Genovesi P. (2018). Which taxa are alien? Criteria, applications, and uncertainties. Bioscience.

[bib26] Forcella F., Wood J. (1984). Colonization potentials of alien weeds are related to their 'native' distributions: implications for plant quarantine. J. Aust. Inst. Agric. Sci..

[bib27] Fowler A.J., Lodge D.M., Hsia J.F. (2007). Failure of the Lacey Act to protect US ecosystems against animal invasions. Front. Ecol. Environ..

[bib28] Gaertner M., Biggs R., Te Beest M. (2014). Invasive plants as drivers of regime shifts: identifying high-priority invaders that alter feedback relationships. Divers. Distrib..

[bib29] Gallardo B., Aldridge D.C., González-Moreno P. (2017). Protected areas offer refuge from invasive species spreading under climate change. Glob. Change Biol..

[bib30] García-de-Lomas J., Vilà M. (2015). Lists of harmful alien organisms: are the national regulations adapted to the global world?. Biol. Invasions.

[bib31] García-Díaz P., Kerezsy A., Unmack P.J. (2018). Transport pathways shape the biogeography of alien freshwater fishes in Australia. Divers. Distrib..

[bib32] Halstead J.M., Michaud J., Hallas-Burt S. (2003). Hedonic analysis of effects of a nonnative invader (*Myriophyllum heterophyllum*) on New Hampshire (USA) lakefront properties. Environ. Manag..

[bib33] Hanspach J., Kühn I., Pyšek P. (2008). Correlates of naturalization and occupancy of introduced ornamentals in Germany. Perpect. Plant Ecol. Evol. Syst..

[bib34] Havel J.E., Kovalenko K.E., Thomaz S.M. (2015). Aquatic invasive species: challenges for the future. Hydrobiologia.

[bib35] Hawkins C.L., Bacher S., Essl F. (2015). Framework and guidelines for implementing the proposed IUCN Environmental Impact Classification for Alien Taxa (EICAT). Divers. Distrib..

[bib36] Hayes K.R., Barry S.C. (2008). Are there any consistent predictors of invasion success?. Biol. Invasions.

[bib37] Holden M.H., Nyrop J.P., Ellner S.P. (2016). The economic benefit of time-varying surveillance effort for invasive species management. J. Appl. Ecol..

[bib38] Hulme P.E. (2006). Beyond control: wider implications for the management of biological invasions. J. Appl. Ecol..

[bib39] Hulme P.E. (2009). Trade, transport and trouble: managing invasive species pathways in an era of globalization. J. Appl. Ecol..

[bib40] Hulme P.E. (2020). Plant invasions in New Zealand: global lessons in prevention, eradication and control. Biol. Invasions.

[bib41] Hussner A., Stiers I., Verhofstad M. (2017). Management and control methods of invasive alien freshwater aquatic plants: a review. Aquat. Bot..

[bib42] Keane R.M., Crawley M.J. (2002). Exotic plant invasions and the enemy release hypothesis. Trends Ecol. Evol..

[bib43] Kopf R.K., Nimmo D.G., Humphries P. (2017). Confronting the risks of large-scale invasive species control. Nat. Ecol. Evol..

[bib44] Krug R., Roura-Pascual N., Richardson D. (2009). Prioritising areas for the management of invasive alien plants in the CFR: different strategies, different priorities?. South Afr. J. Bot..

[bib45] Kumschick S., Bacher S., Dawson W. (2012). A conceptual framework for prioritization of invasive alien species for management according to their impact. NeoBiota.

[bib46] Latombe G., Pyšek P., Jeschke J.M. (2017). A vision for global monitoring of biological invasions. Biol. Conserv..

[bib47] Leung B., Lodge D.M., Finnoff D. (2002). An ounce of prevention or a pound of cure: bioeconomic risk analysis of invasive species. Proc. R. Soc. Lond. B-Biol. Sci..

[bib48] Li F.F., Liu X.Y., Zhu J.F. (2022). The role of genetic factors in the differential invasion success of two *Spartina* species in China. Front. Plant Sci..

[bib49] Li F.F., Gao K.X., Oduor A.M. (2024). High-throughput DNA sequencing identifies population genetic structure and signatures of local adaptation in invasive populations of *Spartina alterniflora* in China. Biol. Invasions.

[bib50] Lieurance D., Canavan S., Behringer D.C. (2023). Identifying invasive species threats, pathways, and impacts to improve biosecurity. Ecosphere.

[bib51] Lonsdale W.M. (1999). Global patterns of plant invasions and the concept of invasibility. Ecology.

[bib52] Lowe S., Browne M., Boudjelas S. (2000). https://www.iucn.org/sites/default/files/2022-04/100_worst_invasive_species_english.pdf.

[bib53] Luque G.M., Bellard C., Bertelsmeier C. (2014). The 100th of the world's worst invasive alien species. Biol. Invasions.

[bib54] Macêdo R.L., Haubrock P.J., Klippel G. (2024). The economic costs of invasive aquatic plants: a global perspective on ecology and management gaps. Sci. Total Environ..

[bib55] Madsen J.D., Bloomfield J.A., Eichler L.W. (1991). The decline of native vegetation under dense Eurasian Watermilfoil Canopies. J. Aquat. Plant Manage..

[bib56] Mathakutha R., Steyn C., le Roux P.C. (2019). Invasive species differ in key functional traits from native and non-invasive alien plant species. J. Veg. Sci..

[bib57] McGeoch M.A., Genovesi P., Bellingham P.J. (2016). Prioritizing species, pathways, and sites to achieve conservation targets for biological invasion. Biol. Invasions.

[bib58] Mitchell C.E., Power A.G. (2003). Release of invasive plants from fungal and viral pathogens. Nature.

[bib59] Molnar J.L., Gamboa R.L., Revenga C. (2008). Assessing the global threat of invasive species to marine biodiversity. Front. Ecol. Environ..

[bib60] Myers J.H., Simberloff D., Kuris A.M. (2000). Eradication revisited: dealing with exotic species. Trends Ecol. Evol..

[bib61] National Invasive Species Council (2016). https://www.doi.gov/sites/doi.gov/files/uploads/2016-2018-nisc-management-plan.pdf.

[bib62] Novoa A., Shackleton R., Canavan S. (2018). A framework for engaging stakeholders on the management of alien species. J. Environ. Manag..

[bib63] Oksanen J., Blanchet F.G., Kindt R. (2010). Package ‘vegan’. Community ecology package. R package version.

[bib64] Packer J.G., Meyerson L.A., Richardson D.M. (2017). Global networks for invasion science: benefits, challenges and guidelines. Biol. Invasions.

[bib65] Pagad S., Genovesi P., Carnevali L. (2018). Introducing the global register of introduced and invasive species. Sci. Data.

[bib66] Pagad S., Bisset S., Genovesi P. (2022). Country compendium of the global register of introduced and invasive species. Sci. Data.

[bib67] Peterson A.T. (2003). Predicting the geography of species' invasions via ecological niche modeling. Q. Rev. Biol..

[bib68] Potgieter L.J., Shrestha N., Cadotte M.W. (2022). Prioritizing sites for terrestrial invasive alien plant management in urban ecosystems. Ecol. Solut. Evid..

[bib69] Pyšek P., Richardson D.M., Pergl J. (2008). Geographical and taxonomic biases in invasion ecology. Trends Ecol. Evol..

[bib70] Pyšek P., Jarošík V., Pergl J. (2009). The global invasion success of Central European plants is related to distribution characteristics in their native range and species traits. Divers. Distrib..

[bib71] Pyšek P., Jarošík V., Hulme P.E. (2012). A global assessment of invasive plant impacts on resident species, communities and ecosystems: the interaction of impact measures, invading species' traits and environment. Glob. Change Biol..

[bib72] Pyšek P., Hulme P.E., Meyerson L.A. (2013). Hitting the right target: taxonomic challenges for, and of, plant invasions. AoB Plants.

[bib73] Pyšek P., Hulme P.E., Simberloff D. (2020). Scientists' warning on invasive alien species. Biol. Rev..

[bib74] Qian H., Ricklefs R.E. (2004). Geographical distribution and ecological conservatism of disjunct genera of vascular plants in eastern Asia and eastern North America. J. Ecol..

[bib75] R Core Team (2022). https://www.R-project.org/.

[bib76] Roy H.E., Peyton J., Aldridge D.C. (2014). Horizon scanning for invasive alien species with the potential to threaten biodiversity in Great Britain. Glob. Change Biol..

[bib77] Roy H.E., Rabitsch W., Scalera R. (2018). Developing a framework of minimum standards for the risk assessment of alien species. J. Appl. Ecol..

[bib78] Russell J.C., Kueffer C. (2019). Island biodiversity in the Anthropocene. Annu. Rev. Environ. Resour..

[bib79] Saffer A., Worm T., Takeuchi Y. (2024). GIataR: a spatio-temporal dataset of global invasive and alien species and their traits. Sci. Data.

[bib80] Seebens H., Essl F., Dawson W. (2015). Global trade will accelerate plant invasions in emerging economies under climate change. Glob. Change Biol..

[bib81] Seebens H., Blackburn T.M., Dyer E.E. (2017). No saturation in the accumulation of alien species worldwide. Nat. Commun..

[bib82] Shackleton R.T., Adriaens T., Brundu G. (2019). Stakeholder engagement in the study and management of invasive alien species. J. Environ. Manag..

[bib83] Simberloff D. (2006). Risk assessments, blacklists, and white lists for introduced species: are predictions good enough to be useful?. Agric. Resour. Econ. Rev..

[bib84] Strauss S.Y., Webb C.O., Salamin N. (2006). Exotic taxa less related to native species are more invasive. Proc. Natl. Acad. Sci. U.S.A..

[bib85] Thompson K., Hodgson J.G., Rich T.C. (1995). Native and alien invasive plants: more of the same?. Ecography.

[bib86] Thouvenot L., Haury J., Thiebaut G. (2013). A success story: water primroses, aquatic plant pests. Conserv. Mar. Freshw. Ecosyst..

[bib95] Turbelin A.J., Malamud B.D., Francis R.A. (2017). Mapping the global state of invasive alien species: patterns of invasion and policy responses. Glob. Ecol. Biogeogr..

[bib87] van Kleunen M., Dawson W., Essl F. (2015). Global exchange and accumulation of non-native plants. Nature.

[bib88] Venette R.C., Gordon D.R., Juzwik J., Poland T.M., Patel-Weynand T., Finch D.M. (2021). Invasive Species in Forests and Rangelands of the United States: A Comprehensive Science Synthesis for the United States Forest Sector.

[bib89] Vilà M., Hulme P.E. (2017).

[bib90] Vilà M., Espinar J.L., Hejda M. (2011). Ecological impacts of invasive alien plants: a meta-analysis of their effects on species, communities and ecosystems. Ecol. Lett..

[bib91] Weber J., Panetta F.D., Virtue J. (2009). An analysis of assessment outcomes from eight years' operation of the Australian border weed risk assessment system. J. Environ. Manag..

[bib92] Wilson J.R., Dormontt E.E., Prentis P.J. (2009). Something in the way you move: dispersal pathways affect invasion success. Trends Ecol. Evol..

[bib93] Winter M., Schweiger O., Klotz S. (2009). Plant extinctions and introductions lead to phylogenetic and taxonomic homogenization of the European flora. Proc. Natl. Acad. Sci. U.S.A..

[bib94] Xu H.G., Qiang S., Han Z.M. (2006). The status and causes of alien species invasion in China. Biodivers. Conserv..

