# Supplementary material for: Global invasive alien plant management lists: Assessing current practices and adapting to new demands
Source: Plant Divers. 2024 Nov 14;47(4):666–80. doi: 10.1016/j.pld.2024.11.002 (PMC12302625; doi:10.1016/j.pld.2024.11.002)
Supplement: Multimedia component 1 [file mmc1.docx]

**Supplemental Information for:**

**Global Invasive Alien Plant Management Lists: Assessing Current Practices and Adapting to New Demands**

Fei-Fei Li^1, 2*^, Qiang Hao^1, 2^, Xia Cui^1, 2^, Ruo-Zhu Lin^3^, Bin-Sheng Luo^4^, Jin-Shuang Ma^1, 2*^

1. Beijing Botanical Garden, Beijing, 100093, China;

2. Key Laboratory of National Forestry and Grassland Administration on Plant Ex situ Conservation, Beijing, 100093, China;

3. Key Laboratory of Forest Protection of the National Forestry and Grassland Administration, Ecology and Nature Conservation Institute, Chinese Academy of Forestry, Beijing 100091, China;

4. Lushan Botanical Garden, Jiangxi Province and Chinese Academy of Sciences, Lushan, 332900, China

**Table S1 The source documents of the lists and related information***

| Country | Release year | Source document of the list | List name | Issuing department | Reference Link |
| --- | --- | --- | --- | --- | --- |
| Argentina | 2021 | Resolution 109/2021 (Resolución 109/2021) | Annex I, List of Invasive, Potentially Invasive and Cryptogenic Alien Species Management Reviews (Anexo I, Lista de Especies Exoticas Invasoras, Potencialmente Invasoras y Criptogénicas Revisiones de Dirección) | Ministry of Environment and Sustainable Development | https://www.boletinoficial.gob.ar/detalleAviso/primera/242964/20210414 |
| Australia | 2021; 1999 (2012 and 2013 updated) | The National Priority List of Exotic Environmental Pests, Weeds and Diseases; Weeds of National Significance | Appendix B: 169 species shortlisted (Phase 2)  for the EEPL, Table B8 Weeds and freshwater algae; Appendix C: The higher risk EEPL species (Phase 3); Weeds of national significance (WONS) | Department of Agriculture, Fisheries and Forestry; Department of Sustainability, Environment, Water, Population and Communities | <https://www.agriculture.gov.au/biosecurity-trade/policy/environmental/priority-list>; Thorp (2001) |
| Canada | 2013 (2019 updated) | Phytosanitary Requirements to Prevent the Introduction of Plants Regulated as Pests in Canada | Appendix 1 Regulated Pest Plants | the Canadian Food Inspection Agency (CFIA) | D-12-01: Phytosanitary Requirements to Prevent the Introduction of Plants Regulated as Pests in Canada - Canadian Food Inspection Agency |
| China | 2023; 2003 (2010, 2014, 2016 updated) | Key Management List of Invasive Alien Species (重点管理外来入侵物种名录); List of Alien Invasive Species in China Natural Ecosystem (1-4 Batch) (中国自然生态系统外来入侵物种名单（第一-四批）) | - | The Ministry of Agriculture and Rural Affairs, the Ministry of Natural Resources, the Ministry of Ecology and Environment, the Ministry of Housing and Urban-Rural Development, the General Administration of Customs, the National Forestry and Grassland administration; the Ministry of Ecology and Environment | <https://www.moa.gov.cn/govpublic/KJJYS/202211/t20221109_6415160.htm>; <https://www.mee.gov.cn/gkml/zj/wj/200910/t20091022_172155.htm>; <https://www.mee.gov.cn/gkml/hbb/bwj/201001/t20100126_184831.htm>; <https://www.mee.gov.cn/gkml/hbb/bgg/201408/t20140828_288367.htm>; <https://www.mee.gov.cn/gkml/hbb/bgg/201612/t20161226_373636.htm> |
| Cook Islands | 2016 | Cook Islands National Invasive Species Strategy and Action Plan 2019 - 2025 | Annex 2, Priority terrestrial invasive species of Cook Islands | Government of Cook Islands | <https://chm.cbd.int/api/v2013/documents/B63E7679-3021-D4C6-9E51-67575CF0AB8E/attachments/Cook%20Is%20NISSAP%202019%20-%2005.pdf> |
| European Union | 2014 (2016, 2017, 2019, 2022 updated) | Regulation (EU) No 1143/2014; Commission Implementing Regulation (EU) 2016/1141;  Commission Implementing Regulation (EU) 2017/1263;  Commission Implementing Regulation (EU) 2019/1262;  Commission Implementing Regulation (EU) 2022/1203 | List of Invasive Alien Species of Union Concern | European Union | <https://eur-lex.europa.eu/eli/reg/2014/1143/oj>; <https://eur-lex.europa.eu/legal-content/EN/TXT/?uri=celex%3A32016R1141>; <https://eur-lex.europa.eu/eli/reg_impl/2017/1263/oj>; <https://eur-lex.europa.eu/eli/reg_impl/2019/1262/oj>; <https://eur-lex.europa.eu/legal-content/EN/TXT/?uri=CELEX%3A32022R1203> |
| Finland | 2019; 2023 | 704/2019, On the Management of Risks Caused by Alien Species (704/2019, Vieraslajeista Aiheutuvien Riskien Hallinnasta); 912/2023, Amending § 2 and Annexes of the Government Decree on the Management of Risks Caused by Alien Species (912/2023, vieraslajeista aiheutuvien riskien hallinnasta annetun valtioneuvoston asetuksen 2 §:n ja liitteiden muuttamisesta) | Appendix B (Liite B) | The State Council | <https://www.finlex.fi/fi/laki/alkup/2019/20190704#Pidm46494958910688>; <https://www.finlex.fi/fi/laki/alkup/2023/20230912> |
| France | 2019; 2020; 2019; 2019; 2019;  2019;  2018 (2020 updated) | Order of 9 August 2019 on the Prevention of The Introduction And Spread Of Invasive Alien Plant Species In Guadeloupe - Prohibition Of All Activities Involving Live Specimens (Arrêté du 9 août 2019 relatif à la prévention de l'introduction et de la propagation des espèces végétales exotiques envahissantes sur le territoire de la Guadeloupe - interdiction de toutes activités portant sur des spécimens vivants);  Order of November 30, 2020 relating to the prevention of the introduction and spread of invasive exotic plant species in the territory of Saint-Martin - ban on all activities involving live specimens (Arrêté du 30 novembre 2020 relatif à la prévention de l'introduction et de la propagation des espèces végétales exotiques envahissantes sur le territoire de Saint-Martin - interdiction de toutes activités portant sur des spécimens vivants);  Order of August 9, 2019 relating to the prevention of the introduction and spread of invasive exotic plant species in the territory of Martinique - ban on all activities involving live specimens (Arrêté du 9 août 2019 relatif à la prévention de l'introduction et de la propagation des espèces végétales exotiques envahissantes sur le territoire de la Martinique - interdiction de toutes activités portant sur des spécimens vivants);  Order of April 1, 2019 relating to the prevention of the introduction and spread of invasive exotic plant species in the territory of Guyana - ban on all activities involving live specimens (Arrêté du 1er avril 2019 relatif à la prévention de l'introduction et de la propagation des espèces végétales exotiques envahissantes sur le territoire de la Guyane - interdiction de toutes activités portant sur des spécimens vivants); Order of April 1, 2019 relating to the prevention of the introduction and spread of invasive exotic plant species in the territory of Reunion - Prohibition of all activities involving live specimens (Arrêté du 1er avril 2019 relatif à la prévention de l'introduction et de la propagation des espèces végétales exotiques envahissantes sur le territoire de La Réunion - Interdiction de toutes activités portant sur des spécimens vivants); Order of September 9, 2019 relating to the regulation of the introduction and spread of invasive exotic plant species in the territory of Mayotte (Arrêté du 9 septembre 2019 relatif à la régulation de l'introduction et de la propagation des espèces végétales exotiques envahissantes sur le territoire de Mayotte); Order of February 14, 2018 relating to the prevention of the introduction and spread of invasive exotic plant species in metropolitan territory (Arrêté du 14 février 2018 relatif à la prévention de l'introduction et de la propagation des espèces végétales exotiques envahissantes sur le territoire métropolitain); Order of March 10, 2020 updating the list of invasive exotic animal and plant species on metropolitan territory (Arrêté du 10 mars 2020 portant mise à jour de la liste des espèces animales et végétales exotiques envahissantes sur le territoire métropolitain) | Appendices (Annexes) | Ministry of Ecological and Inclusive Transition, Ministry of Agriculture and Food; Ministry of Ecological and Inclusive Transition, Ministry of Agriculture and Food; Ministry of Ecological and Inclusive Transition, Ministry of Agriculture and Food; Ministry of Ecological and Inclusive Transition, Ministry of Agriculture and Food; Ministry of Ecological and Inclusive Transition, Ministry of Agriculture and Food, Ministry of Overseas Territories; Ministry of Ecological and Inclusive Transition, Ministry of Agriculture and Food | <https://www.legifrance.gouv.fr/jorf/id/JORFTEXT000039196385/>; <https://www.legifrance.gouv.fr/jorf/id/JORFTEXT000042738214>; <https://www.legifrance.gouv.fr/loda/id/JORFTEXT000039126771/>; <https://www.legifrance.gouv.fr/loda/id/JORFTEXT000038424487/>; <https://www.legifrance.gouv.fr/loda/id/JORFTEXT000038358649/>; <https://www.legifrance.gouv.fr/loda/id/JORFTEXT000039183621/>; <https://www.legifrance.gouv.fr/loda/id/JORFTEXT000036629837/>;  <https://www.legifrance.gouv.fr/jorf/id/JORFTEXT000041875937/> |
| Hungary | 2012; 2007; 2008 | 86/2012. (VIII. 15.) VM Decree on the commercial collection and marketing of fodder plant seed mixtures intended for the preservation of the natural environment (86/2012. (VIII. 15.) VM rendelet a természetes környezet megőrzésére szánt takarmánynövény-vetőmagkeverékek kereskedelmi célú begyűjtéséről és forgalmazásáról); 269/2007. (X. 18.) Government decree on land use rules for the maintenance of NATURA 2000 grassland areas (269/2007. (X. 18.) Korm. rendelet a NATURA 2000 gyepterületek fenntartásának földhasználati szabályairól); 346/2008. (XII. 30.) Government decree on the protection of woody plants (346/2008. (XII. 30.) Korm. rendelet a fás szárú növények védelméről) | Annex 3, List of invasive (*) and other weed-like plant species posing a threat from a nature conservation perspective (3. melléklet, A természetvédelmi szempontból veszélyt jelentő, inváziós- (*) és egyéb gyom jellegű növényfajok listája); Annex, Soft and woody invasive and non-native plant species that threaten species and habitats of community importance occurring in Natura 2000 areas (Melléklet, A Natura 2000 területeken előforduló közösségi jelentőségű fajokat és élőhelyeket veszélyeztető lágy- és fásszárú inváziós és termőhely-idegen növényfajok); Annex 1, Invasive woody plants (1. melléklet, Inváziós fajú fás szárú növények) | Ministry of Rural Development; Government | <https://net.jogtar.hu/jogszabaly?docid=a1200086.vm>; <https://net.jogtar.hu/jogszabaly?docid=a0700269.kor>; <https://net.jogtar.hu/jogszabaly?docid=a0800346.kor> |
| Japan | 2005 (2006, 2007, 2008, 2010, 2011, 2013, 2014, 2015, 2016, 2018, 2020, 2023 updated) | List of living organisms regulated based on the Invasive Alien Species Act (特定外来生物による生態系等に係る被害の防止に関する法律に基づき規制される生物のリスト) | List of Regulated Living Organisms under the Invasive Alien Species Act [Plant Kingdom] | Ministry of the Environment | https://www.env.go.jp/nature/intro/2outline/list.html |
| Kenya | 2013 | The Wildlife Conservation and Management Act, 2013 (No. 47 of 2013), SEVENTH SCHEDULE (s.49(1)) | National List of Invasive Species | National Government of Kenya | https://kenyalaw.org/kl/fileadmin/pdfdownloads/Acts/WildlifeConservationandManagement%20Act2013.pdf |
| Korea | 2023 | Notification of designation of ecosystem disturbing organisms, No. 2023-228 (행정규칙/생태계교란생물지정고시) | - | Ministry of Environment | https://law.go.kr/%ED%96%89%EC%A0%95%EA%B7%9C%EC%B9%99/%EC%83%9D%ED%83%9C%EA%B3%84%EA%B5%90%EB%9E%80%EC%83%9D%EB%AC%BC%EC%A7%80%EC%A0%95%EA%B3%A0%EC%8B%9C |
| Malta | 2020 | National Strategy for Preventing and Mitigating the Impact of Invasive Alien Species (IAS) in the Maltese Islands | Table 3, Terrestrial Alien Species with invasive categories 3.5, 4, 4.5 and 5 | The Environment and Resources Authority (ERA) | ERA (2020) |
| Mexico | 2016 | Agreement By Which The List Of Invasive Exotic Species For Mexico Is Determined (Acuerdo Por El Que Se Determina La Lista De Las Especies Exóticas Invasoras Para México) | List of Invasive Exotic Species for Mexico (Lista de Especies Exóticas Invasoras para México) | Secretariat of Environment and Natural Resources (SEMARNAT) | <https://www.dof.gob.mx/nota_detalle.php?codigo=5464456&fecha=07/12/2016#gsc.tab=0> |
| New Zealand | 2016 | Biosecurity (Notifiable Organisms) Order 2016; This order is made under section 45(2) of the Biosecurity Act 1993 on the advice and with the consent of the Executive Council. | Schedule Notifiable organisms, Invasive plants | Order in Council | <https://www.legislation.govt.nz/regulation/public/2016/0073/9.0/whole.html> |
| Poland | 2022 | REGULATION OF THE COUNCIL OF MINISTERS of 9 December 2022 on the list of invasive alien species posing a threat to the Union and the list of invasive alien species posing a threat to Poland, remedial actions and measures aimed at restoring the natural state of ecosystems (ROZPORZĄDZENIE RADY MINISTRÓW z dnia 9 grudnia 2022 r. w sprawie listy inwazyjnych gatunków obcych stwarzających zagrożenie dla Unii i listy inwazyjnych gatunków obcych stwarzających zagrożenie dla Polski, działań zaradczych oraz środków mających na celu przywrócenie naturalnego stanu ekosystemów) | List of invasive alien species posing a threat to Poland (Lista inwazyjnych gatunków obcych stwarzających zagrożenie dla Polski) | The Council of Ministers | https://isap.sejm.gov.pl/isap.nsf/DocDetails.xsp?id=WDU20220002649 |
| Rwanda | 2016 | Study to assess the impacts of invasive alien species (Flowering plants, fish and insects) in natural forests, agro-ecosystems, lakes and wetland ecosystems in Rwanda and develop their management plans | Invasive alien plant species of Rwanda | Rwanda Environment Management Authority (REMA) | BIOCEM-RD Ltd (Biodiversity Conservation and Development) (2016) |
| Slovak Republic | 2020 | Government Regulation of the Slovak Republic No. 449/2019 Coll. (Nariadenie vlády Slovenskej republiky č. 449/2019 Z. z.) | List of invasive alien species of concern to the Slovak Republic (ZOZNAM INVÁZNYCH NEPÔVODNÝCH DRUHOV RASTLÍN VZBUDZUJÚCICH OBAVY SLOVENSKEJ REPUBLIKY) | Government of the Slovak Republic (Vláda Slovenskej republiky) | https://www.slov-lex.sk/pravne-predpisy/SK/ZZ/2019/449/20200101 |
| South Africa | 2020 | National Environmental Management: Biodiversity Act, 2004 (ACT NO. 10 OF 2004) | Alien and Invasive Species Lists, 2020 | Department of Environment, Forestry and Fisheries | https://www.gov.za/documents/national-environmental-management-biodiversity-act-0 |
| Spain | 2013; 2019; 2020; 2023 | Royal Decree 630/2013, of August 2, which regulates the Spanish Catalog of invasive exotic species (Real Decreto 630/2013, de 2 de agosto, por el que se regula el Catálogo español de especies exóticas invasoras); Royal Decree 216/2019, of March 29, which approves the list of invasive exotic species of concern for the outermost region of the Canary Islands and which modifies Royal Decree 630/2013, of August 2, by which regulates the Spanish Catalog of invasive exotic species (Real Decreto 216/2019, de 29 de marzo, por el que se aprueba la lista de especies exóticas invasoras preocupantes para la región ultraperiférica de las islas Canarias y por el que se modifica el Real Decreto 630/2013, de 2 de agosto, por el que se regula el Catálogo español de especies exóticas invasoras); Order TED/1126/2020, of November 20, which modifies the Annex to Royal Decree 139/2011, of February 4, for the development of the List of Wild Species under Special Protection Regime and the Spanish Catalog of Species Threatened, and the Annex to Royal Decree 630/2013, of August 2, which regulates the Spanish Catalog of Invasive Exotic Species (Orden TED/1126/2020, de 20 de noviembre, por la que se modifica el Anexo del Real Decreto 139/2011, de 4 de febrero, para el desarrollo del Listado de Especies Silvestres en Régimen de Protección Especial y del Catálogo Español de Especies Amenazadas, y el Anexo del Real Decreto 630/2013, de 2 de agosto, por el que se regula el Catálogo Español de Especies Exóticas Invasoras); Order TED/339/2023, of March 30, which modifies the annex to Royal Decree 139/2011, of February 4, for the development of the List of Wild Species under Special Protection Regime and the Spanish Catalog of Species Threatened, and the annex to Royal Decree 630/2013, of August 2, which regulates the Spanish Catalog of Invasive Exotic Species (Orden TED/339/2023, de 30 de marzo, por la que se modifica el anexo del Real Decreto 139/2011, de 4 de febrero, para el desarrollo del Listado de Especies Silvestres en Régimen de Protección Especial y del Catálogo Español de Especies Amenazadas, y el anexo del Real Decreto 630/2013, de 2 de agosto, por el que se regula el Catálogo Español de Especies Exóticas Invasoras) | Attachment (Anexo); Attachment, Five. The annex is modified as follows: List of invasive alien species of concern for the outermost region of the Canary Islands (ANEXO, Cinco. Se modifica el anexo de la siguiente forma; Lista de especies exóticas invasoras preocupantes para la región ultraperiférica de las islas Canarias); Second article. Modification of the annex to Royal Decree 630/2013, of August 2, which regulates the Spanish Catalog of Invasive Exotic Species (Artículo segundo. Modificación del anexo del Real Decreto 630/2013, de 2 de agosto, que regula el Catálogo Español de Especies Exóticas Invasoras); Second article.Modification of the annex to Royal Decree 630/2013, of August 2, which regulates the Spanish Catalog of Invasive Exotic Species (Artículo segundo. Modificación del anexo del Real Decreto 630/2013, de 2 de agosto, que regula el Catálogo Español de Especies Exóticas Invasoras.) | Ministry of Agriculture, Food and Environment; Ministry for the Ecological Transition; Ministry for the Ecological Transition and the Demographic Challenge; Ministry for the Ecological Transition and the Demographic Challenge | <https://www.boe.es/eli/es/rd/2013/08/02/630/con>; <https://www.boe.es/eli/es/rd/2019/03/29/216/con>; <https://www.boe.es/eli/es/o/2020/11/20/ted1126>; <https://www.boe.es/eli/es/o/2023/03/30/ted339> |
| Switzerland | 2022 | Alien species in Switzerland, overview of alien species and their impacts. Status 2022 (Gebietsfremde Arten in der Schweiz, Übersicht über die gebietsfremden Arten und ihre Auswirkungen. Stand 2022) | Species list (Artenliste) | Federal Office for the Environment | https://www.bafu.admin.ch/bafu/en/home/topics/biodiversity/publications-studies/publications/invasive-alien-species.html |
| Thailand | 2018 | Measures for Prevention, Control and Eradication of Alien Species (เรื่อง มาตรการป้องกัน ควบคุม และกำาจัดชนิดพันธุ์ต่างถิ่น) | List 1: Invasive alien species that have already invaded; List 2: Alien species with a tendency to invade; List 3: Alien species with a history of invasion in other countries but not yet invaded in Thailand; List 4: Invasive alien species that have not yet entered Thailand  (รายการ 1 ชนิดพันธุ์ต่างถิ่นที่รุกรานแล้ว; รายการ 2 ชนิดพันธุ์ต่างถิ่นที่มีแนวโน้มรุกราน; รายการ 3 ชนิดพันธุ์ต่างถิ่นที่มีประวัติว่ารุกรานแล้วในประเทศอื่นแต่ยังไม่รุกรานในประเทศไทย; รายการ 4 ชนิดพันธุ์ต่างถิ่นที่รุกรานที่ยังไม่เข้ามาในประเทศไทย) | Office of Natural Resources and Environmental Policy and Planning | https://chm-thai.onep.go.th/?page_id=2258 |
| United States | 2004 (2010 updated) | PART 360—Noxious Weed Regulations | § 360.200 Designation of noxious weeds. | The Code of Federal Regulations (CFR) | https://www.ecfr.gov/current/title-7/subtitle-A/part-2/subpart-C/section-2.22 |
| Uruguay | 2015 | Invasive exotic species in Uruguay (Especies ex'ticas invasorasen el Uruguay) | - | National Committee on Invasive Exotic Species | https://www.gub.uy/ministerio-ambiente/comunicacion/publicaciones/lista-especies-exoticas-invasoras-uruguay |

* The titles of the original documents and list names have been translated into English, with the original language versions provided in parentheses where applicable.

BIOCEM-RD Ltd (Biodiversity Conservation, E. M. a. R., & Development). (2016). *Study to assess the impacts of invasive alien species (Flowering plants, fish and insects) in natural forests, agro-ecosystems, lakes and wetland ecosystems in Rwanda and develop their management plans*.

ERA. (2020). *National strategy for preventing and mitigating the impact of Invasive Alien Species (IAS) in the Maltese Islands : measures for implementation - 2020*. Environment & Resources Authority.

Thorp, J. (2001). The determination of weeds of national significance. *National Weeds Strategy Executive Committee*, **234**.

**Table S3. Proportion of invasive alien plants (IAPs) in each country's lists compared to the total number of introduced and invasive plants in GRIIS for each country/** **union**

| Country | Number of list IAPs | Total number of introduced and invasive plants | Proportion of number of list IAPs to Total (%) |
| --- | --- | --- | --- |
| Argentina | 407 | 475 | 85.68 |
| Australia | 53 | 2630 | 2.02 |
| Canada | 21 | 1249 | 1.68 |
| China | 42 | 448 | 9.38 |
| Cook Islands | 17 | 429 | 3.96 |
| European Union | 40 | 6461 | 0.62 |
| Finland | 17 | 58 | 29.31 |
| France | 356 | 1684 | 21.14 |
| Hungary | 31 | 74 | 41.89 |
| Japan | 19 | 1483 | 1.28 |
| Kenya | 12 | 208 | 5.77 |
| Korea | 15 | 34 | 44.12 |
| Malta | 46 | 165 | 27.88 |
| Mexico | 134 | 595 | 22.52 |
| New Zealand | 10 | 517 | 1.93 |
| Poland | 7 | 890 | 0.79 |
| Rwanda | 32 | 239 | 13.39 |
| Slovakia | 7 | 856 | 0.82 |
| South Africa | 378 | 1436 | 26.32 |
| Spain | 76 | 612 | 12.42 |
| Switzerland | 93 | 399 | 23.31 |
| Thailand | 107 | 116 | 92.24 |
| United States | 109 | 3837 | 2.84 |
| Uruguay | 19 | 57 | 33.33 |
| Total | 1247 | 7846 | 15.89 |

**Table S6. Poisson regression results for listing frequency by habitats and growth forms of invasive alien plants (IAPs)**

| Habitats and  growth forms | Listing frequency | Estimate | Std. Error | z value | P value |
| --- | --- | --- | --- | --- | --- |
| Aquatic | 2.57 | 0.94 | 0.08 | 12.37 | < 2e-16 |
| Aquatic or Terrestrial | 1.65 | -0.44 | 0.18 | -2.46 | 0.01 |
| Terrestrial | 1.56 | -0.49 | 0.08 | -6.19 | 5.86e-10 |
| Epiphyte | 1.00 | -5.792e-14 | 0.32 | 0.00 | 1.00 |
| Herb | 1.61 | 0.48 | 0.32 | 1.51 | 0.13 |
| Liana | 1.44 | 0.36 | 0.34 | 1.08 | 0.28 |
| Parasitic | 1.50 | 0.41 | 0.43 | 0.95 | 0.34 |
| Shrub | 1.82 | 0.60 | 0.32 | 1.87 | 0.06 |
| Tree | 1.55 | 0.44 | 0.32 | 1.39 | 0.16 |
| Vine | 1.80 | 0.59 | 0.33 | 1.76 | 0.08 |

**Table S7. Similarities of invasive alien plants (IAPs) listed in national management inventories among countries**

| Country | Fr-AF | Fr | Fr-NA | Fr-SA | Ar | Au | Ca | Ch | Co | Eu | Fi | Hu | Ja | Ke | Ko | Ma | Me | Po | Rw | Sl | SA | Sw | Th | USA | Ur | NZ | Sp-AF | Sp |
| --- | --- | --- | --- | --- | --- | --- | --- | --- | --- | --- | --- | --- | --- | --- | --- | --- | --- | --- | --- | --- | --- | --- | --- | --- | --- | --- | --- | --- |
| Fr-AF | 1.00 | 0.11 | 0.17 | 0.10 | 0.04 | 0.05 | 0.01 | 0.03 | 0.03 | 0.11 | 0.00 | 0.02 | 0.02 | 0.02 | 0.00 | 0.04 | 0.06 | 0.01 | 0.05 | 0.00 | 0.15 | 0.07 | 0.09 | 0.03 | 0.01 | 0.01 | 0.03 | 0.05 |
| Fr | 0.11 | 1.00 | 0.26 | 0.39 | 0.00 | 0.09 | 0.05 | 0.06 | 0.02 | 0.91 | 0.00 | 0.07 | 0.11 | 0.06 | 0.02 | 0.04 | 0.04 | 0.00 | 0.03 | 0.00 | 0.03 | 0.16 | 0.06 | 0.02 | 0.00 | 0.06 | 0.00 | 0.14 |
| Fr-NA | 0.17 | 0.26 | 1.00 | 0.18 | 0.01 | 0.06 | 0.03 | 0.04 | 0.03 | 0.26 | 0.01 | 0.04 | 0.05 | 0.02 | 0.03 | 0.03 | 0.07 | 0.01 | 0.03 | 0.01 | 0.08 | 0.10 | 0.08 | 0.02 | 0.01 | 0.03 | 0.03 | 0.09 |
| Fr-SA | 0.10 | 0.39 | 0.18 | 1.00 | 0.00 | 0.08 | 0.04 | 0.05 | 0.04 | 0.43 | 0.00 | 0.06 | 0.08 | 0.02 | 0.00 | 0.01 | 0.04 | 0.00 | 0.03 | 0.00 | 0.02 | 0.12 | 0.03 | 0.02 | 0.00 | 0.02 | 0.02 | 0.11 |
| Ar | 0.04 | 0.00 | 0.01 | 0.00 | 1.00 | 0.01 | 0.01 | 0.00 | 0.00 | 0.00 | 0.01 | 0.02 | 0.01 | 0.01 | 0.01 | 0.02 | 0.08 | 0.00 | 0.01 | 0.01 | 0.10 | 0.03 | 0.02 | 0.01 | 0.03 | 0.00 | 0.01 | 0.01 |
| Au | 0.05 | 0.09 | 0.06 | 0.08 | 0.01 | 1.00 | 0.07 | 0.08 | 0.03 | 0.09 | 0.00 | 0.00 | 0.06 | 0.03 | 0.00 | 0.01 | 0.03 | 0.02 | 0.04 | 0.00 | 0.04 | 0.04 | 0.07 | 0.06 | 0.03 | 0.05 | 0.04 | 0.06 |
| Ca | 0.01 | 0.05 | 0.03 | 0.04 | 0.01 | 0.07 | 1.00 | 0.00 | 0.03 | 0.03 | 0.00 | 0.00 | 0.03 | 0.00 | 0.00 | 0.02 | 0.04 | 0.00 | 0.00 | 0.00 | 0.01 | 0.03 | 0.01 | 0.06 | 0.03 | 0.00 | 0.02 | 0.01 |
| Ch | 0.03 | 0.06 | 0.04 | 0.05 | 0.00 | 0.08 | 0.00 | 1.00 | 0.04 | 0.07 | 0.02 | 0.04 | 0.07 | 0.06 | 0.10 | 0.00 | 0.01 | 0.00 | 0.06 | 0.04 | 0.03 | 0.06 | 0.09 | 0.01 | 0.00 | 0.04 | 0.02 | 0.07 |
| Co | 0.03 | 0.02 | 0.03 | 0.04 | 0.00 | 0.03 | 0.03 | 0.04 | 1.00 | 0.02 | 0.00 | 0.00 | 0.03 | 0.04 | 0.00 | 0.03 | 0.01 | 0.00 | 0.02 | 0.00 | 0.02 | 0.00 | 0.03 | 0.02 | 0.00 | 0.00 | 0.00 | 0.00 |
| Eu | 0.11 | 0.91 | 0.26 | 0.43 | 0.00 | 0.09 | 0.03 | 0.07 | 0.02 | 1.00 | 0.00 | 0.08 | 0.11 | 0.06 | 0.02 | 0.04 | 0.04 | 0.00 | 0.03 | 0.00 | 0.03 | 0.17 | 0.06 | 0.02 | 0.00 | 0.06 | 0.00 | 0.13 |
| Fi | 0.00 | 0.00 | 0.01 | 0.00 | 0.01 | 0.00 | 0.00 | 0.02 | 0.00 | 0.00 | 1.00 | 0.12 | 0.00 | 0.00 | 0.03 | 0.00 | 0.01 | 0.20 | 0.00 | 0.09 | 0.01 | 0.05 | 0.01 | 0.00 | 0.00 | 0.00 | 0.00 | 0.03 |
| Hu | 0.02 | 0.07 | 0.04 | 0.06 | 0.02 | 0.00 | 0.00 | 0.04 | 0.00 | 0.08 | 0.12 | 1.00 | 0.02 | 0.00 | 0.02 | 0.01 | 0.02 | 0.12 | 0.00 | 0.15 | 0.02 | 0.13 | 0.01 | 0.01 | 0.02 | 0.00 | 0.00 | 0.06 |
| Ja | 0.02 | 0.11 | 0.05 | 0.08 | 0.01 | 0.06 | 0.03 | 0.07 | 0.03 | 0.11 | 0.00 | 0.02 | 1.00 | 0.03 | 0.03 | 0.00 | 0.01 | 0.00 | 0.02 | 0.00 | 0.01 | 0.04 | 0.04 | 0.02 | 0.03 | 0.04 | 0.00 | 0.06 |
| Ke | 0.02 | 0.06 | 0.02 | 0.02 | 0.01 | 0.03 | 0.00 | 0.06 | 0.04 | 0.06 | 0.00 | 0.00 | 0.03 | 1.00 | 0.00 | 0.02 | 0.02 | 0.00 | 0.13 | 0.00 | 0.03 | 0.01 | 0.04 | 0.00 | 0.00 | 0.10 | 0.00 | 0.05 |
| Ko | 0.00 | 0.02 | 0.03 | 0.00 | 0.01 | 0.00 | 0.00 | 0.10 | 0.00 | 0.02 | 0.03 | 0.02 | 0.03 | 0.00 | 1.00 | 0.02 | 0.01 | 0.00 | 0.00 | 0.05 | 0.01 | 0.05 | 0.01 | 0.00 | 0.00 | 0.00 | 0.00 | 0.03 |
| Ma | 0.04 | 0.04 | 0.03 | 0.01 | 0.02 | 0.01 | 0.02 | 0.00 | 0.03 | 0.04 | 0.00 | 0.01 | 0.00 | 0.02 | 0.02 | 1.00 | 0.03 | 0.00 | 0.05 | 0.00 | 0.04 | 0.01 | 0.03 | 0.00 | 0.02 | 0.00 | 0.06 | 0.07 |
| Me | 0.06 | 0.04 | 0.07 | 0.04 | 0.08 | 0.03 | 0.04 | 0.01 | 0.01 | 0.04 | 0.01 | 0.02 | 0.01 | 0.02 | 0.01 | 0.03 | 1.00 | 0.01 | 0.02 | 0.00 | 0.06 | 0.04 | 0.06 | 0.07 | 0.02 | 0.01 | 0.03 | 0.05 |
| Po | 0.01 | 0.00 | 0.01 | 0.00 | 0.00 | 0.02 | 0.00 | 0.00 | 0.00 | 0.00 | 0.20 | 0.12 | 0.00 | 0.00 | 0.00 | 0.00 | 0.01 | 1.00 | 0.00 | 0.00 | 0.01 | 0.03 | 0.03 | 0.00 | 0.04 | 0.00 | 0.03 | 0.02 |
| Rw | 0.05 | 0.03 | 0.03 | 0.03 | 0.01 | 0.04 | 0.00 | 0.06 | 0.02 | 0.03 | 0.00 | 0.00 | 0.02 | 0.13 | 0.00 | 0.05 | 0.02 | 0.00 | 1.00 | 0.00 | 0.05 | 0.00 | 0.09 | 0.01 | 0.00 | 0.05 | 0.02 | 0.05 |
| Sl | 0.00 | 0.00 | 0.01 | 0.00 | 0.01 | 0.00 | 0.00 | 0.04 | 0.00 | 0.00 | 0.09 | 0.15 | 0.00 | 0.00 | 0.05 | 0.00 | 0.00 | 0.00 | 0.00 | 1.00 | 0.00 | 0.04 | 0.00 | 0.00 | 0.00 | 0.00 | 0.00 | 0.02 |
| SA | 0.15 | 0.03 | 0.08 | 0.02 | 0.10 | 0.04 | 0.01 | 0.03 | 0.02 | 0.03 | 0.01 | 0.02 | 0.01 | 0.03 | 0.01 | 0.04 | 0.06 | 0.01 | 0.05 | 0.00 | 1.00 | 0.04 | 0.08 | 0.02 | 0.02 | 0.01 | 0.04 | 0.06 |
| Sw | 0.07 | 0.16 | 0.10 | 0.12 | 0.03 | 0.04 | 0.03 | 0.06 | 0.00 | 0.17 | 0.05 | 0.13 | 0.04 | 0.01 | 0.05 | 0.01 | 0.04 | 0.03 | 0.00 | 0.04 | 0.04 | 1.00 | 0.03 | 0.03 | 0.01 | 0.02 | 0.00 | 0.12 |
| Th | 0.09 | 0.06 | 0.08 | 0.03 | 0.02 | 0.07 | 0.01 | 0.09 | 0.03 | 0.06 | 0.01 | 0.01 | 0.04 | 0.04 | 0.01 | 0.03 | 0.06 | 0.03 | 0.09 | 0.00 | 0.08 | 0.03 | 1.00 | 0.05 | 0.01 | 0.04 | 0.04 | 0.05 |
| USA | 0.03 | 0.02 | 0.02 | 0.02 | 0.01 | 0.06 | 0.06 | 0.01 | 0.02 | 0.02 | 0.00 | 0.01 | 0.02 | 0.00 | 0.00 | 0.00 | 0.07 | 0.00 | 0.01 | 0.00 | 0.02 | 0.03 | 0.05 | 1.00 | 0.01 | 0.02 | 0.02 | 0.02 |
| Ur | 0.01 | 0.00 | 0.01 | 0.00 | 0.03 | 0.03 | 0.03 | 0.00 | 0.00 | 0.00 | 0.00 | 0.02 | 0.03 | 0.00 | 0.00 | 0.02 | 0.02 | 0.04 | 0.00 | 0.00 | 0.02 | 0.01 | 0.01 | 0.01 | 1.00 | 0.04 | 0.05 | 0.01 |
| NZ | 0.01 | 0.06 | 0.03 | 0.02 | 0.00 | 0.05 | 0.00 | 0.04 | 0.00 | 0.06 | 0.00 | 0.00 | 0.04 | 0.10 | 0.00 | 0.00 | 0.01 | 0.00 | 0.05 | 0.00 | 0.01 | 0.02 | 0.04 | 0.02 | 0.04 | 1.00 | 0.00 | 0.03 |
| Sp-AF | 0.03 | 0.00 | 0.03 | 0.02 | 0.01 | 0.04 | 0.02 | 0.02 | 0.00 | 0.00 | 0.00 | 0.00 | 0.00 | 0.00 | 0.00 | 0.06 | 0.03 | 0.03 | 0.02 | 0.00 | 0.04 | 0.00 | 0.04 | 0.02 | 0.05 | 0.00 | 1.00 | 0.03 |
| Sp | 0.05 | 0.14 | 0.09 | 0.11 | 0.01 | 0.06 | 0.01 | 0.07 | 0.00 | 0.13 | 0.03 | 0.06 | 0.06 | 0.05 | 0.03 | 0.07 | 0.05 | 0.02 | 0.05 | 0.02 | 0.06 | 0.12 | 0.05 | 0.02 | 0.01 | 0.03 | 0.03 | 1.00 |

Fr: France, Ar: Argentina, Au: Australia, Ca: Canada, Ch: China, Co: Cook Islands, Eu: European Union, Fi: Finland, Hu: Hungary, Ja: Japan, Ke: Kenya, Ko: Korea, Ma: Malta, Me: Mexico, Po: Poland, Rw: Rwanda, Sl: Slovakia, SA: South Africa, Sw: Switzerland, Th: Thailand, USA: United States, Ur: Uruguay, NZ: New Zealand, Sp: Spain, NA = North America, SA = South America, AF = Africa. Notations combining a country name with a continent abbreviation represent that country’s overseas territories in the respective continent.

**Table S8 Intercontinental exchange patterns of invasive alien plants (IAPs): Number of species by native and invaded continents**

| Invaded continents | IAPs native continents | No. of IAPs |
| --- | --- | --- |
| Africa | Aisa | 203 |
| Africa | North America | 192 |
| Africa | South America | 173 |
| Africa | Africa | 126 |
| Africa | Oceania | 107 |
| Africa | Europe | 85 |
| Aisa | North America | 92 |
| Aisa | South America | 81 |
| Aisa | Africa | 40 |
| Aisa | Aisa | 37 |
| Aisa | Europe | 26 |
| Aisa | Oceania | 11 |
| Europe | North America | 90 |
| Europe | Aisa | 83 |
| Europe | South America | 48 |
| Europe | Africa | 43 |
| Europe | Europe | 29 |
| Europe | Oceania | 17 |
| Oceania | North America | 43 |
| Oceania | South America | 42 |
| Oceania | Africa | 32 |
| Oceania | Aisa | 25 |
| Oceania | Europe | 17 |
| Oceania | Oceania | 7 |
| North America | Aisa | 190 |
| North America | Africa | 152 |
| North America | South America | 96 |
| North America | Europe | 85 |
| North America | North America | 69 |
| North America | Oceania | 68 |
| South America | Aisa | 329 |
| South America | Europe | 297 |
| South America | Africa | 253 |
| South America | North America | 72 |
| South America | Oceania | 41 |
| South America | South America | 35 |

**
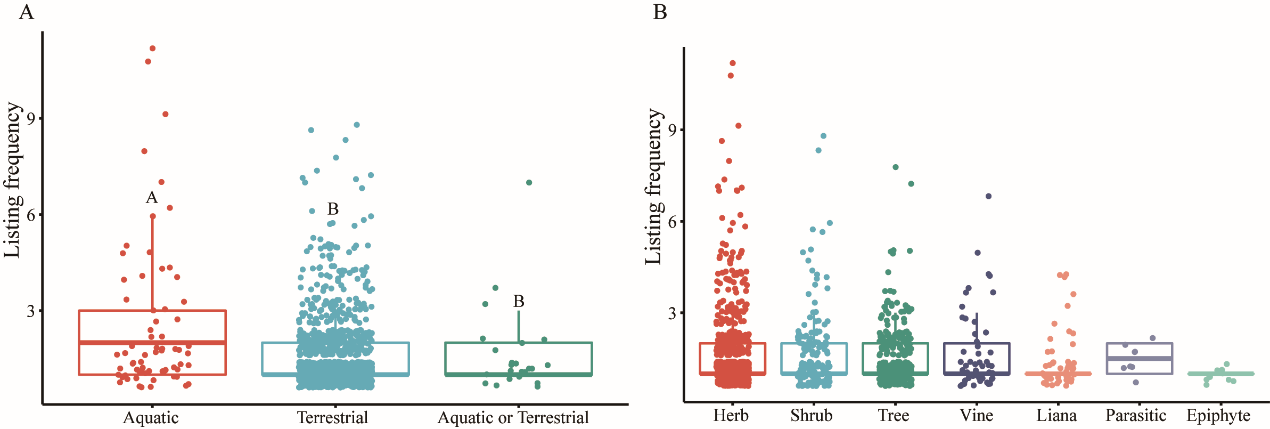
**

**Fig. S1. Distribution of listing frequency for invasive alien plants (IAPs) by habitat (A) and growth forms (B).**


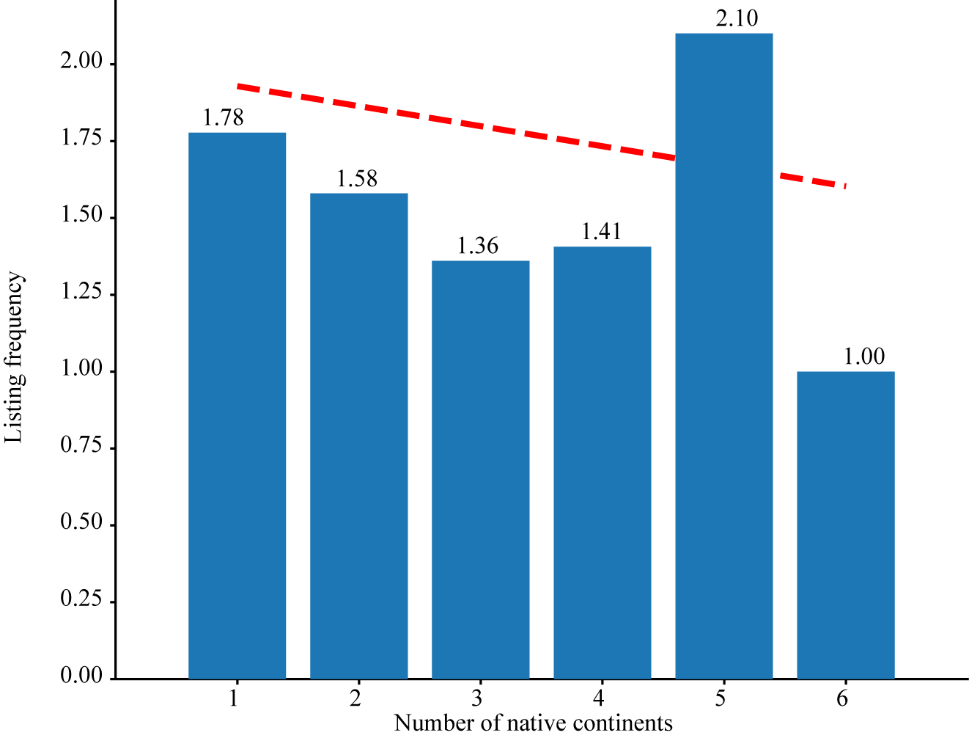


**Fig. S2. Relationship between native distribution range and listing frequency of invasive alien plants (IAPs).**

**
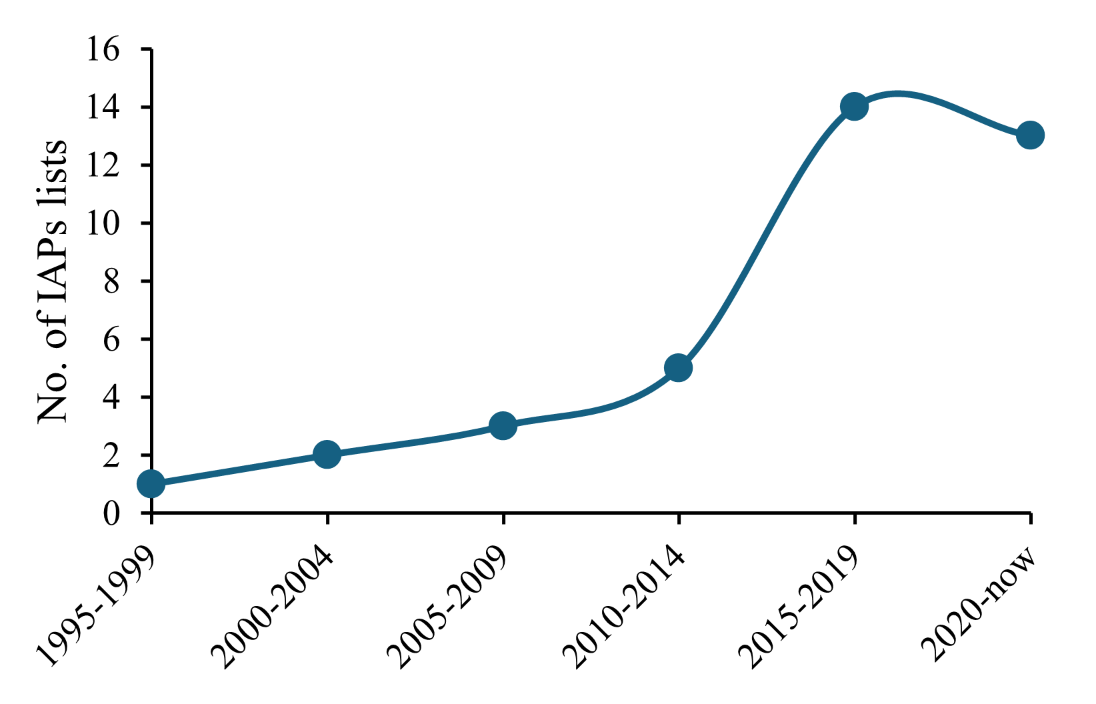
**

**Fig. S3. Cumulative growth in the number of invasive alien species (IAS) national management lists.**

**
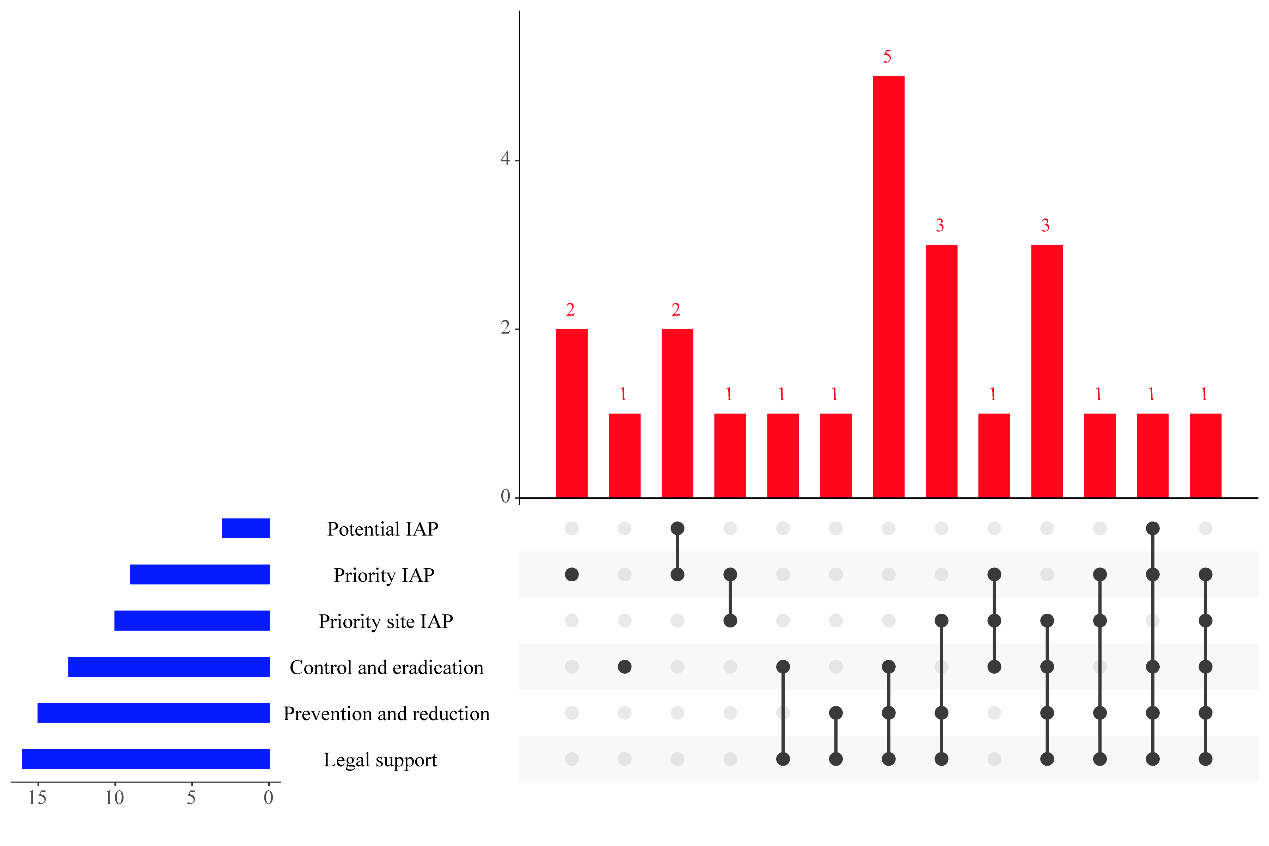
**

**Fig. S4. Upset plot for framework analysis of invasive alien plants (IAPs) lists characteristics and management regulations across countries. Lower left is the size of each mainly management characteristic. Lower right is the intersections, black points with intersections and gray points with no intersections.**


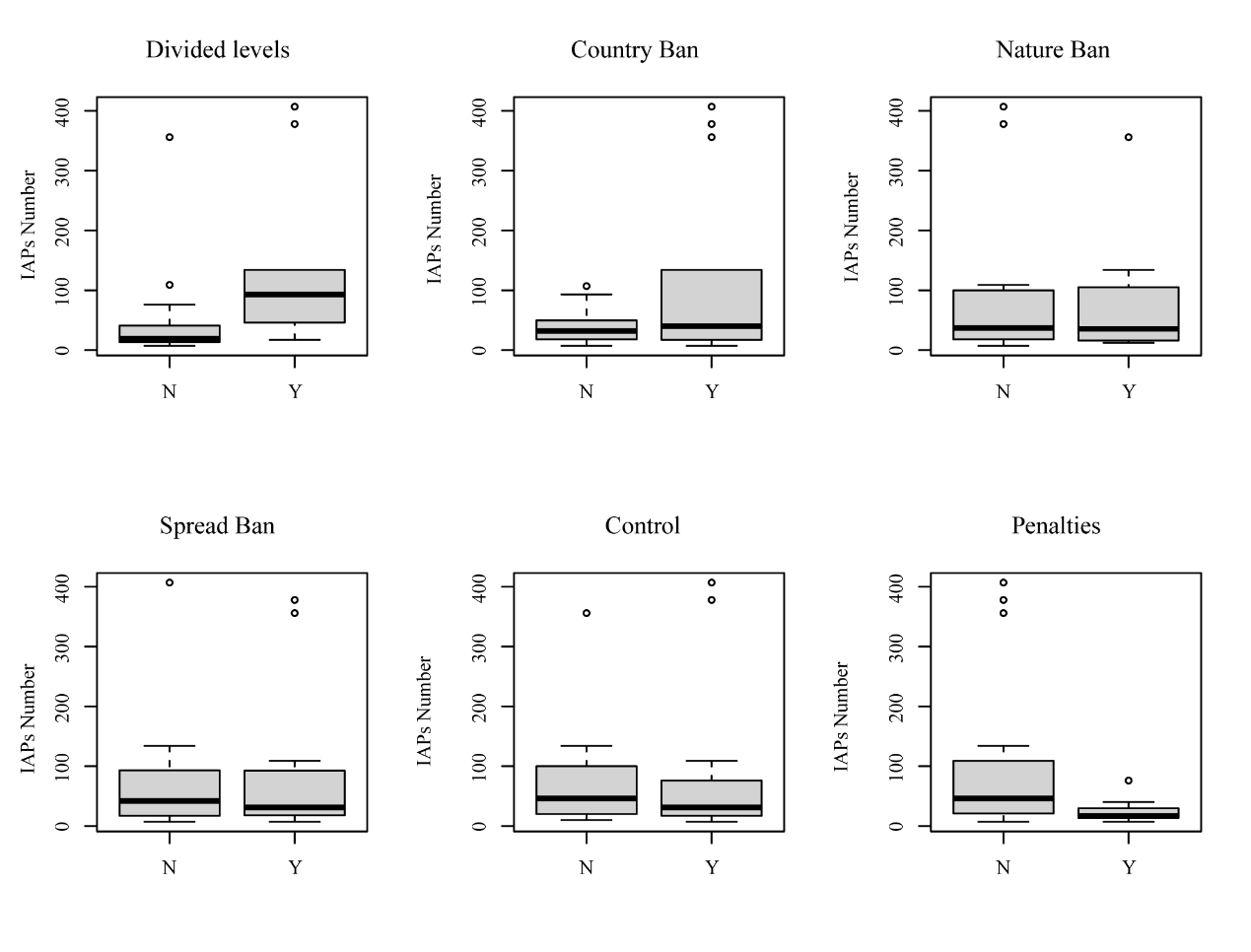


**Fig. S5. Impact of management measures on invasive alien plants (IAPs) list size**
